# Role of autonomic receptors in ethyl ferulate-induced cardiovascular effects in normotensive and hypertensive female rats

**DOI:** 10.1007/s00424-026-03170-3

**Published:** 2026-04-25

**Authors:** Francisca Valdirene de S. Nunes, Samuel S. P. Araujo, Juliana A. da Silva, José Guilherme V. de Assunção, Ariell A. de Oliveira, Carlos E. R. Dourado, Vandercley de O. Damasceno, Aldeídia P. de Oliveira, Maria do Carmo C. Martins, Mayara C. de Morais, Damião P. de Sousa, Thyago M. de Queiroz, Renato N. Soriano, Luiz G. S. Branco, Helio C. Salgado, João Paulo J. Sabino

**Affiliations:** 1 Department of Biophysics and Physiology, Federal University of Piauí, Ininga, Teresina, PI 64049-550 Brazil; 2 Department of Pharmaceutical Sciences, Federal University of Paraíba, João Pessoa-PB, Brazil; 3 Academic Center of Vitória, Federal University of Pernambuco, Vitória de Santo Antão-PE, Brazil; 4 Division of Physiology and Biophysics, Life Sciences Institute, Federal University of Juiz de Fora-campus GV, Governador Valadares-MG, Brazil; 5 Department of Basic and Oral Biology, Ribeirão Preto School of Dentistry, University of São Paulo, Ribeirão Preto-SP, Brazil; 6 Department of Physiology, Medical School of Ribeirão Preto, University of São Paulo, Ribeirão Preto-SP, Brazil

**Keywords:** Ethyl Ferulate, Female rats, Wistar, SHR, Hypotension, Bradycardia

## Abstract

**Aims:**

Ethyl ferulate (EF) may offer novel benefits in hypertension. We investigated the contribution of autonomic receptors to EF-induced effects in female Wistar and SHR.

**Methods:**

Anesthetized females received EF intravenously at 7.5, 15, or 30 mg/kg. Autonomic involvement was evaluated using pretreatment with atropine, atenolol, or hexamethonium. MAP and HR were continuously monitored.

**Results:**

EF induced rapid and pronounced reductions in MAP and HR in both strains. In Wistar rats, muscarinic and nicotinic blockade significantly attenuated these responses. In SHR, atropine abolished bradycardia and partially mitigated hypotension, whereas β1-adrenergic and nitric oxide synthase inhibition had minimal impact.

**Conclusion:**

EF acutely lowers MAP and HR, with muscarinic and nicotinic receptors driving the effects in normotensive females, and muscarinic pathways partially mediating responses in hypertensive females. These results position EF as a compelling candidate for antihypertensive therapy and underscore the critical need to study cardiovascular interventions in female hypertension models.

**Supplementary Information:**

The online version contains supplementary material available at 10.1007/s00424-026-03170-3.

## Introduction

Arterial hypertension (AH) is the most prevalent chronic noncommunicable disease and is characterized by its multifactorial nature. It is widely recognized as a major contributor to the development of cardiovascular diseases (CVD), kidney disorders, and stroke, as well as a leading cause of high morbidity and premature mortality [1, 2]. The pathogenesis of AH is complex, as the regulation of blood pressure (BP) under physiological conditions requires the integrated actions of the renin–angiotensin–aldosterone system (RAAS), the sympathetic nervous system (SNS), vascular remodeling and stiffness, nitric oxide (NO) production, and the involvement of both the immune system and sex hormones, particularly estradiol [3–5].

Estradiol is a key hormone throughout the female reproductive lifespan and also plays an important role in BP regulation, influencing the prevalence of hypertension in women. During the reproductive phase, women exhibit a significantly lower risk of developing systemic arterial hypertension (SAH) compared with age- and health-matched men [3, 6–8]. However, this lower prevalence has contributed to a limited number of studies specifically addressing the mechanisms underlying BP regulation in women [9]. Therefore, studies encompassing the female reproductive period are essential to elucidate BP regulatory pathways and to support the development or refinement of therapeutic strategies for SAH.

Among experimental models of SAH, spontaneously hypertensive rats (SHR) are particularly noteworthy. Although hypertensive, female SHR exhibit less cardiovascular damage than males of the same strain [10], suggesting that sex hormones exert cardioprotective effects. Thus, experimental data obtained from female SHR may provide a valuable foundation for the development of therapeutic strategies specifically targeted at women with SAH.

In recent years, inflammation has gained increasing attention due to its association with both the development and maintenance of hypertension, as chronic low-grade inflammation is frequently observed in this condition. Given the involvement of the immune system and inflammatory responses as key mediators in the pathogenesis of hypertension [11–14], compounds with anti-inflammatory properties may hold therapeutic potential for hypertension and its complications. One such candidate is ethyl ferulate (EF), a phenylpropanoid widely distributed in plants, particularly in grains such as corn and rice [15–17].

The anti-inflammatory activity of EF has been described by Wu and coworkers, who demonstrated that EF significantly inhibited prostaglandin E2 (PGE2) production in LPS-stimulated RAW 264.7 macrophages in a concentration-dependent manner. In the same study, EF also reduced levels of IL-6, TNF-α, and oxidative stress in vitro. In vivo, pretreatment with EF markedly attenuated leukocyte infiltration, as well as TNF-α and IL-6 levels, in LPS-induced lung injury in mice [18]. More recently, Zeng and colleagues demonstrated the cardioprotective and antifibrotic effects of EF in mice subjected to myocardial infarction (MI). In this context, EF inhibited cardiac fibroblast proliferation and collagen deposition by suppressing TGFBR1 signaling during myocardial fibrosis [19].

Although EF has demonstrated anti-inflammatory and cardioprotective effects, its potential therapeutic impact on SAH in female SHR remains unexplored. Therefore, this study aimed to assess the effects of EF on cardiovascular parameters in female SHR and to elucidate the contribution of autonomic receptors and nitric oxide pathways to the cardiovascular responses induced by this compound.

## Methods

### Animal experimentation

Experiments were conducted on 12-week-old female Wistar and SHR rats, weighing 190–250 g. Animals were housed in the Animal Facility of the Department of Biophysics and Physiology, Federal University of Piauí (UFPI), under controlled temperature (23 ± 1 °C) and a 12:12 h light–dark cycle. Rats had free access to standard chow and water. All experiments were performed between 09:00 and 16:00 in the Laboratory for the Study of Reflex Control of Arterial Pressure and Pulmonary Ventilation (LEPAVE), Department of Biophysics and Physiology, UFPI. The study was conducted in accordance with the guidelines of the National Council for Animal Experimentation (CONCEA) and approved by the Ethics Committee on Animal Use of UFPI (CEUA/UFPI, protocol #804/2023). At the end of the experiments, animals were euthanized with an overdose of sodium thiopental (150 mg/kg, i.v.).

### Preparation of EF

EF was dissolved in a vehicle consisting of 1% dimethyl sulfoxide (DMSO), 2% Tween 20, and 0.9% sodium chloride solution. For the evaluation of its acute effects, EF was administered intravenously at doses of 7.5 mg/kg, 15 mg/kg, and 30 mg/kg, adapted from a previous study¹⁶. The synthesis method of EF employed in the experimental protocols has been previously described [20–23].

### Vaginal cytology collection and analysis in Wistar and SHR females

On the day of femoral artery and vein cannulation, vaginal epithelial cells were collected using a dropper containing approximately 0.5 mL of 0.9% sodium chloride solution. The tip of the pipette was carefully inserted into the vaginal orifice of the animal to a depth of approximately 5–10 mm. The saline solution was then gently introduced and aspirated two to three times to collect the cellular material.

After washing, a small drop of the sample was uniformly spread onto a glass slide to obtain a thin smear. The slides were examined under a light microscope (PZO-Labmex, 07021, Studar Lab), initially using a 10× objective and subsequently with a 40× objective without a condenser, following the method described in a previous study²⁴. Based on the determination of the estrous cycle phase, only females in the metestrus and diestrus stages were selected for inclusion in the study.

### Surgical procedure for femoral artery and vein cannulation

Immediately after vaginal cytology analysis, and 48 h prior to the experimental protocols, the females were anesthetized with a mixture of ketamine (75 mg/kg; União Farmacêutica Química S/A, Embu-Guaçu, SP, Brazil) and xylazine (10 mg/kg, i.p.; Hertape Calier Animal Health S/A, Juatuba, MG, Brazil). Polyethylene (PE) cannulas were then implanted in the left femoral artery and vein, made by fusing a PE-10 segment (Intramedic, Becton Dickinson and Company, Sparks, MD, USA) to a PE-50 segment. The arterial cannula was used for direct recording of pulsatile arterial pressure (PAP), while the venous cannula was used for drug administration. The cannulas were exteriorized dorsally and fixed to the nape of the animal. Following surgery, the females were housed individually in recovery cages during the postoperative period.

### Evaluation of the acute effect of EF on cardiovascular parameters in Wistar and SHR females

On the day of the experiment, the arterial catheter was connected to a pressure transducer (MLT0380/D, ADInstruments, Sydney, Australia). The signal, after proper amplification (ML110 Amplifier, ADInstruments, Sydney, Australia), was digitized through an analog-to-digital interface (ML866, ADInstruments, Sydney, Australia) and sampled at 2000 Hz on a microcomputer. After a 60-min adaptation period, sodium nitroprusside (NPS; 8 µg/kg, i.v.; Sigma-Aldrich, St. Louis, MO, USA) was administered to confirm venous cannulation. A baseline recording (30 min) was then performed. Subsequently, the females received four intravenous administrations at 20-min intervals in the following order: (1) vehicle (0.1 mL/100 g); (2) EF 7.5 mg/kg; (3) EF 15 mg/kg; and (4) EF 30 mg/kg. At the end of the experiment, sodium nitroprusside (NPS; 8 µg/kg, i.v.) was again administered to confirm that the intravenous cannula remained viable throughout the procedure.

During the experimental protocols, systolic arterial pressure (SAP), diastolic arterial pressure (DAP), mean arterial pressure (MAP), and heart rate (HR) were derived from PAP. MAP was calculated using the following equation inserted into the LabChart program: 1/3 (SAP) + 2/3 (DAP). The mean MAP and HR values were quantified every 5 s and expressed as delta (Δ; difference between baseline and treatment with the substances). Additionally, the maximum response of the cardiovascular parameters after compound administration was also evaluated.

### Involvement of autonomic receptors and NO in the mechanism of action of EF in Wistar and SHR females

Forty-eight hours after femoral artery and vein cannulation, females of both strains were subjected to blockade of the main autonomic receptors, while only SHR females received the nitric oxide synthesis inhibitor. Following the adaptation period and baseline recording, one group from each strain received one of the following: atropine (2 mg/kg; muscarinic receptor antagonist), atenolol (2 mg/kg; β1-adrenergic receptor antagonist), hexamethonium (20 mg/kg; ganglionic blocker), or L-NAME (3 mg/kg; nitric oxide synthase inhibitor — administered only in SHR). Fifteen minutes after administration of the antagonists, and five minutes in the case of the L-NAME group, the animals received the three doses of EF at 20-min intervals.

### Statistical analysis

Results are expressed as mean ± SEM. Statistical analyses were performed using GraphPad Prism (version 8.0.1) and SigmaPlot (version 11.0). Baseline values and maximal responses of MAP and HR to EF were compared using the unpaired Student’s t-test. Changes in MAP and HR induced by EF over 60 s were analyzed using two-way repeated-measures ANOVA followed by Tukey’s post hoc test. A p value < 0.05 was considered statistically significant.

## Results

### Baseline cardiovascular parameters

Table 1 presents the baseline cardiovascular parameters in Wistar and SHR females used in the study. SHR females exhibited significantly higher systolic arterial pressure (SAP), diastolic arterial pressure (DAP), and MAP (*p* = 0.001) and lower HR compared with Wistar females (*p* < 0.001).

**Table 1:** Baseline values of systolic blood pressure (SBP), diastolic blood pressure (DBP), mean arterial pressure (MAP), and heart rate (HR) in Wistar and SHR females. Data are expressed as mean ± SEM. *p < 0.001 when compared with Wistar group

	SBP (mmHg)		DBP (mmHg)	MAP (mmHg)	HR (bpm)
WISTAR (*n* = 27)		126 ± 2	96 ± 2	106 ± 2	425 ± 6
SHR (*n* = 28)		189 ± 5*	140 ± 3*	156 ± 3*	321 ± 13*

### Acute effect of EF on cardiovascular parameters

**Fig. 1:**
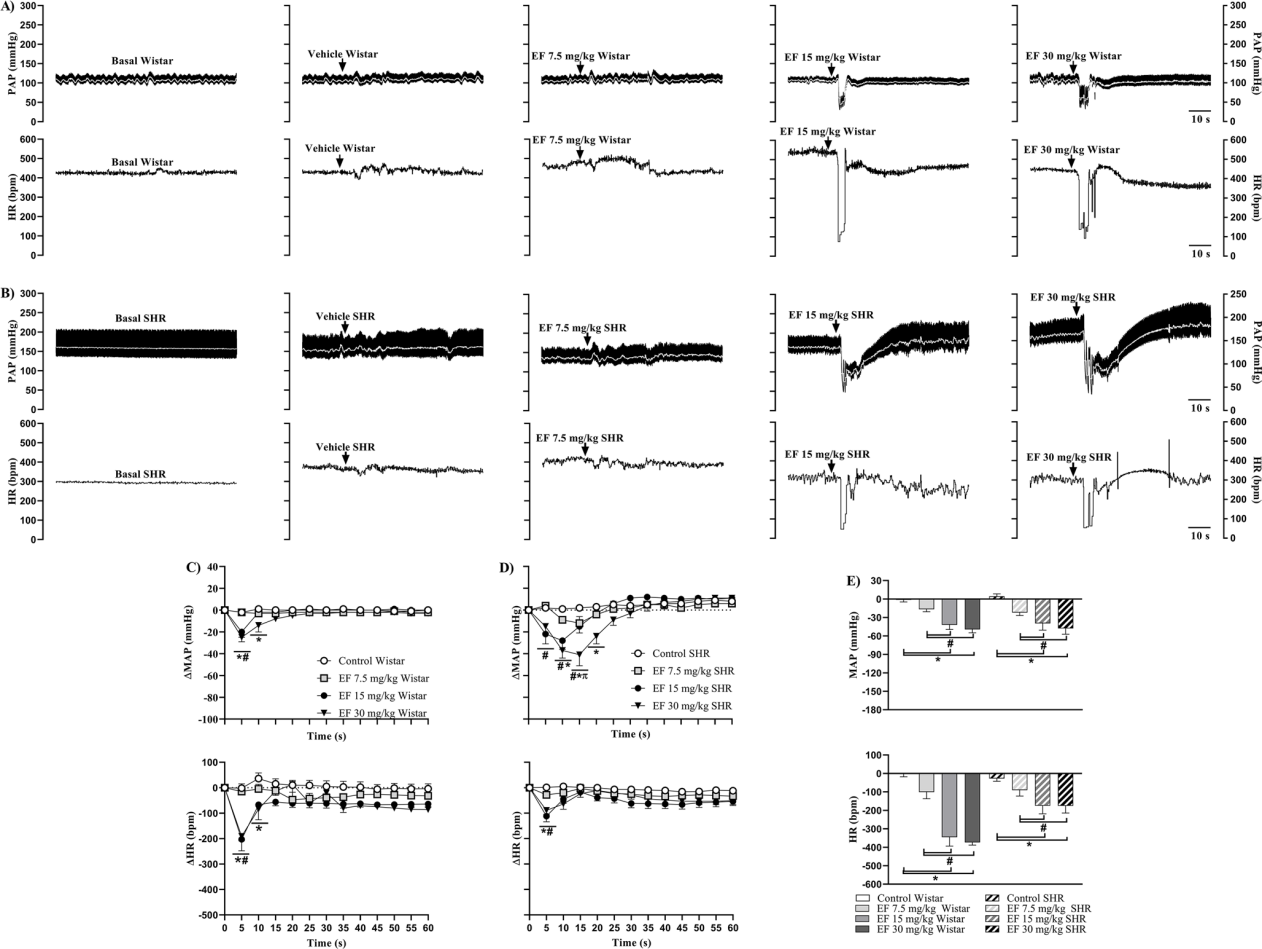
Representative recordings of pulsatile arterial pressure (PAP; upper panel, black trace), MAP (upper panel, white trace), and HR (lower panel) in Wistar (**A**) and SHR (**B**) females under basal conditions, treated with vehicle or EF. Panels C (Wistar) and D (SHR) show changes in MAP (upper panel) and HR (lower panel) induced by vehicle or EF. Panel E shows maximum responses of MAP (upper panel) and HR (lower panel) in Wistar and SHR females treated with vehicle or EF. Data are expressed as mean ± SEM. *p < 0.05 vs. control; #*p* < 0.05 vs. 7.5 mg/kg; πp < 0.05 vs. 15 mg/kg

Figure 1 shows representative recordings of pulsatile arterial pressure (PAP) (upper panel, black trace), MAP (upper panel, white trace), and HR (lower panel) in Wistar (A) and SHR (B) females under baseline conditions and following treatment with vehicle or EF. Additionally, the effects of EF on MAP and HR over 60 s are presented for Wistar (C) and SHR (D) females, along with maximal responses to EF in both strains (E).

In Wistar females, the 15 mg/kg and 30 mg/kg doses of EF induced a significant reduction in MAP and HR compared with the control group and the 7.5 mg/kg dose (Fig. 1C). Similarly, in SHR females, the 15 and 30 mg/kg doses of EF markedly decreased MAP and HR compared with vehicle and 7.5 mg/kg dose groups (Fig. 1D). However, in both strains, the cardiovascular parameters returned to baseline levels one minute after intravenous administration (Supplementary Tables 1 and Supplementary Fig. 1).

Analysis of maximal responses revealed that in both Wistar and SHR females, only the 15 mg/kg and 30 mg/kg doses produced a pronounced reduction in MAP and HR compared with their respective controls and the 7.5 mg/kg dose.

### Acute effect of EF after muscarinic blockade

**Fig. 2:**
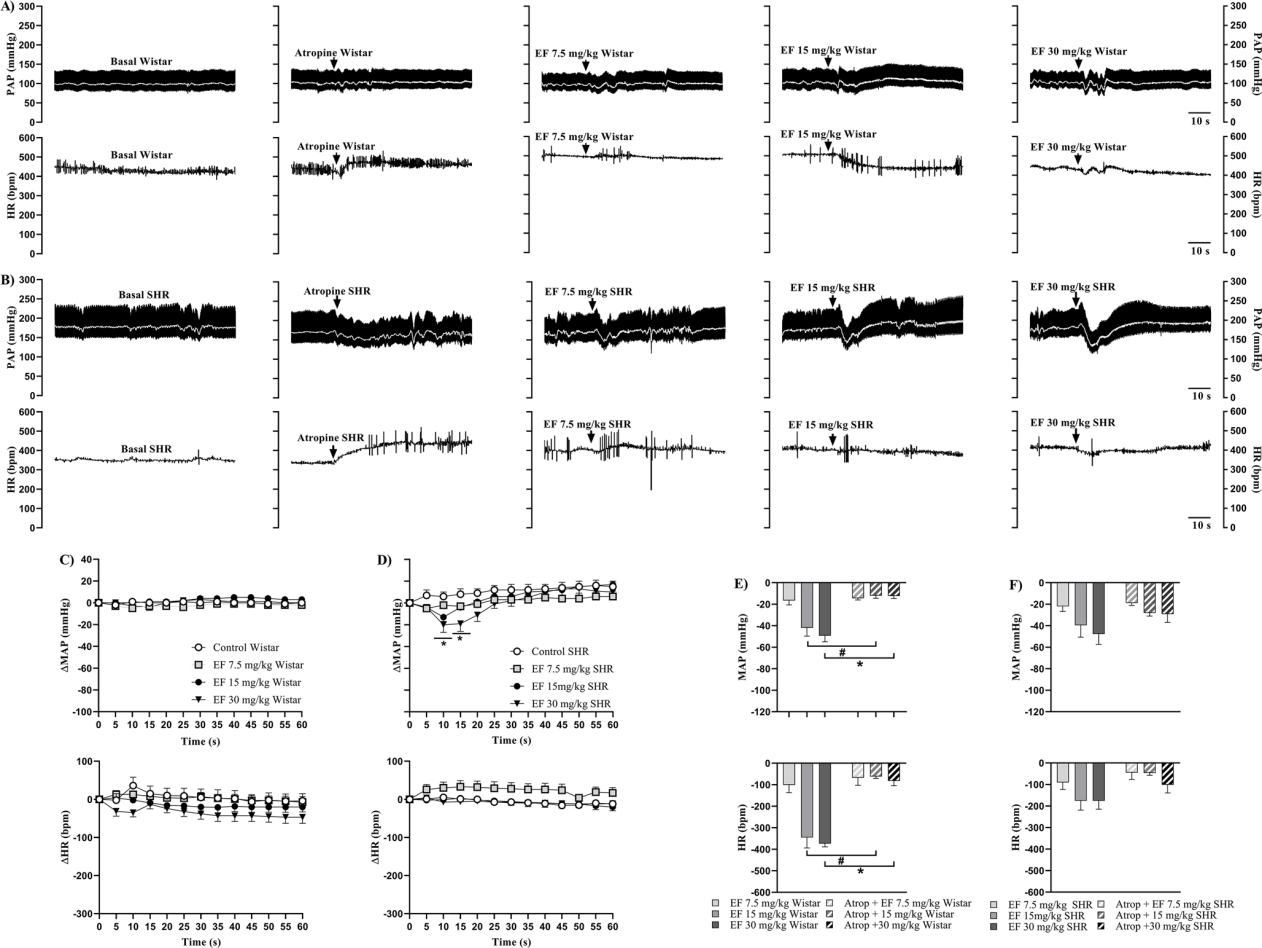
Representative recordings of PAP (upper panel, black trace), MAP (upper panel, white trace), and HR (lower panel) in Wistar (**A**) and SHR (**B**) females under basal conditions, with or without pre-treatment with atropine, followed by EF administration. Panels C (Wistar) and D (SHR) show changes in MAP (upper panel) and HR (lower panel) induced by EF, with or without atropine pre-treatment. Panels E (Wistar) and F (SHR) show maximum responses of MAP (upper panel) and HR (lower panel) induced by EF, with or without atropine pre-treatment. Data are expressed as mean ± SEM. *p < 0.05 vs. control

Figure 2 shows representative recordings of PAP (upper panel, black trace), MAP (upper panel, white trace), and HR (lower panel) in Wistar (A) and SHR (B) females under baseline conditions, after atropine administration, and subsequently treated with EF. Additionally, the effects of EF on MAP and HR over 60 s are presented for Wistar (C) and SHR (D) females, along with maximal cardiovascular responses to EF in Wistar (E) and SHR (F) females, pretreated or not with atropine.

In Wistar females, pretreatment with atropine prevented the 15 and 30 mg/kg doses of EF from inducing significant reductions in MAP and HR (Fig. 2C). Similarly, in SHR females, atropine completely abolished bradycardia and partially prevented hypotension at doses of 15 mg/kg and 30 mg/kg of EF (Fig. 2D). Additionally, in both strains, the cardiovascular parameters returned to baseline levels one minute after the last administration (Supplementary Tables 2 and Supplementary Fig. 2).

Analysis of maximal responses showed that muscarinic blockade in Wistar females prevented the 15 mg/kg and 30 mg/kg doses of EF from decreasing MAP and HR compared with EF administered alone (Fig. 2E). In SHR females, there was also a trend toward attenuation of maximal hypotensive and bradycardic responses, but without statistical significance (Fig. 2F).

### Acute effect of EF after β1-adrenergic blockade

**Fig. 3:**
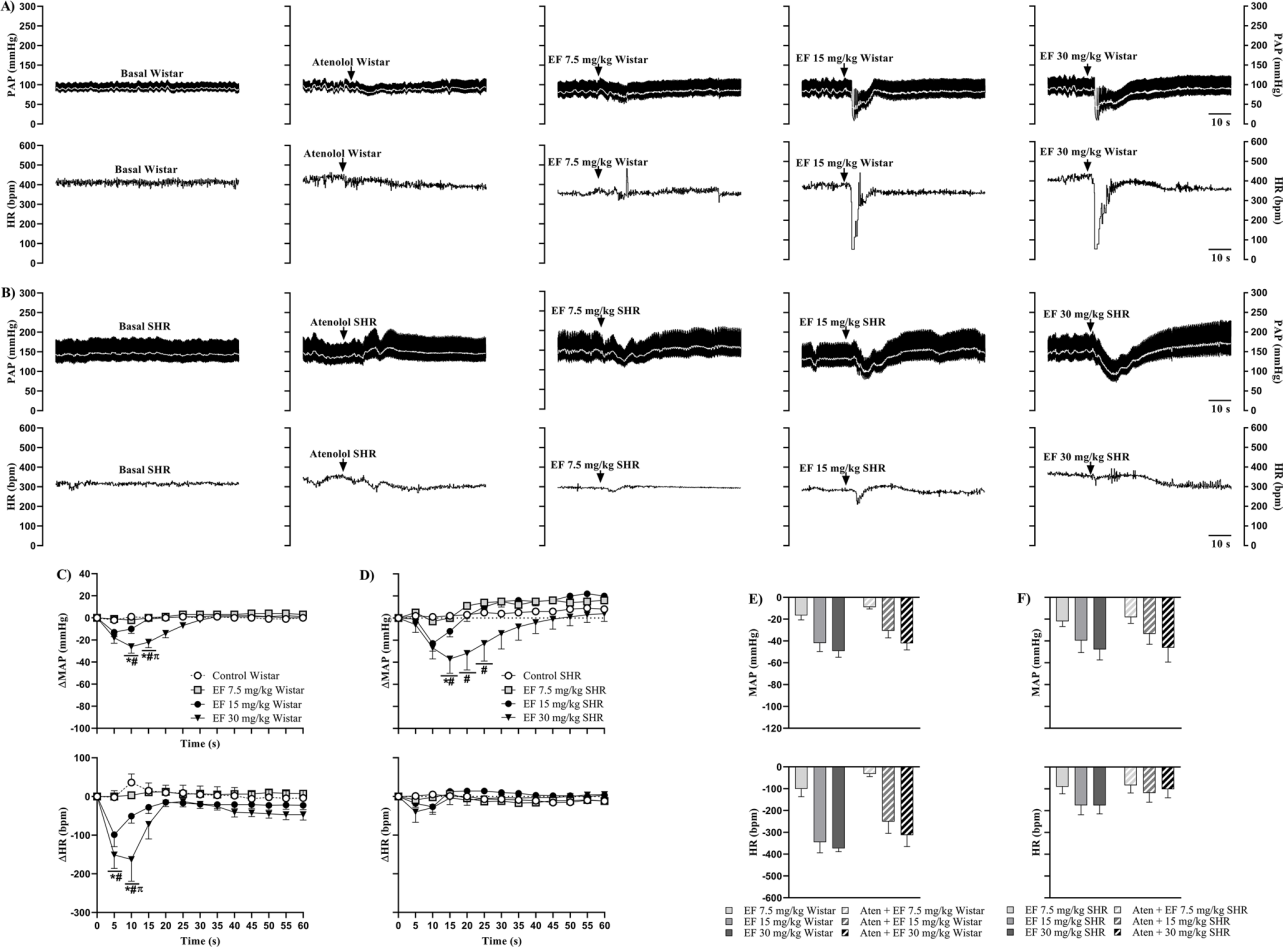
Representative recordings of PAP (upper panel, black trace), MAP (upper panel, white trace), and HR (lower panel) in Wistar (**A**) and SHR (**B**) females under basal conditions, with or without pre-treatment with atenolol, followed by EF administration. Panels C (Wistar) and D (SHR) show changes in MAP (upper panel) and HR (lower panel) induced by EF, with or without atenolol pre-treatment. Panels E (Wistar) and F (SHR) show maximum responses of MAP (upper panel) and HR (lower panel) induced by EF, with or without atenolol pre-treatment. Data are expressed as mean ± SEM. *p < 0.05 vs. control; #*p* < 0.05 vs. 7.5 mg/kg; πp < 0.05 vs. 15 mg/kg

Figure 3 shows representative recordings of PAP (upper panel, black trace), MAP (upper panel, white trace), and HR (lower panel) in Wistar (A) and SHR (B) females under baseline conditions, after atenolol administration, and subsequently treated with EF. Additionally, the effects of EF on MAP and HR over 60 s are presented for Wistar (C) and SHR (D) females, along with maximal cardiovascular responses to EF in Wistar (E) and SHR (F) females, pretreated or not with atenolol.

Pretreatment with atenolol did not prevent the reductions in MAP and HR induced by the 15 and 30 mg/kg doses of EF in Wistar females (Fig. 3C). Similarly, in SHR females, β1-adrenergic blockade did not prevent the decrease in MAP but abolished bradycardia (Fig. 3D). Additionally, in both strains, cardiovascular parameters returned to baseline levels one minute after the last administration (Supplementary Tables 3 and Supplementary Fig. 3).

Analysis of maximal cardiovascular responses revealed that β1-adrenergic blockade did not significantly attenuate the maximal reductions in MAP and HR induced by EF in either Wistar (Fig. 3E) or SHR females (Fig. 3F).

### Acute effect of EF after nicotinic blockade

**Fig. 4:**
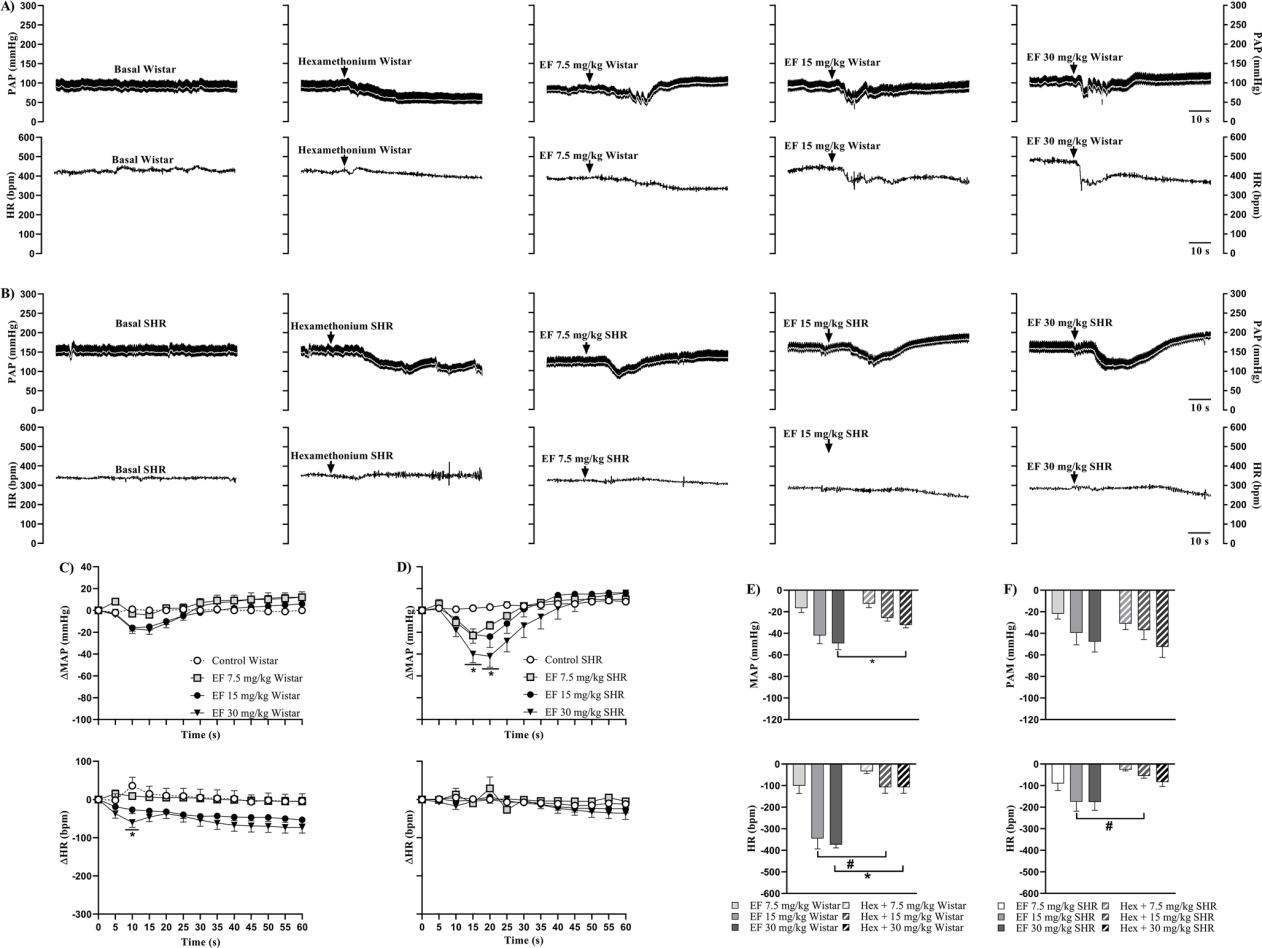
Representative recordings of PAP (upper panel, black trace), MAP (upper panel, white trace), and HR (lower panel) in Wistar (**A**) and SHR (**B**) females under basal conditions, with or without pre-treatment with hexamethonium, followed by EF administration. Panels C (Wistar) and D (SHR) show changes in MAP (upper panel) and HR (lower panel) induced by EF, with or without hexamethonium pre-treatment. Panels E (Wistar) and F (SHR) show maximum responses of MAP (upper panel) and HR (lower panel) induced by EF, with or without hexamethonium pre-treatment. Data are expressed as mean ± SEM. *p < 0.05 vs. control

Figure 4 shows representative recordings of PAP (upper panel, black trace), MAP (upper panel, white trace), and HR (lower panel) in Wistar (A) and SHR (B) females under baseline conditions, after hexamethonium administration, and subsequently treated with EF. Additionally, the effects of EF on MAP and HR over 60 s are presented for Wistar (C) and SHR (D) females, along with maximal cardiovascular responses to EF in Wistar (E) and SHR (F) females, pretreated or not with hexamethonium.

In Wistar females, hexamethonium blocked hypotension and bradycardia induced by the 7.5 and 15 mg/kg doses of EF, except at 30 mg/kg, where moderate bradycardia was observed at 10 s. In contrast, in SHR females, ganglionic blockade amplified the reduction in MAP at all three doses but completely prevented the attenuation of HR following EF administration. Additionally, in both strains, cardiovascular parameters returned to baseline levels one minute after the last administration (Supplementary Tables 4 and Supplementary Fig. 4).

Analysis of maximal responses revealed that in Wistar females, hexamethonium attenuated the reductions in MAP and HR induced by EF when administered alone. In SHR females, however, nicotinic blockade did not significantly affect cardiovascular parameters, except for the 15 mg/kg dose, which attenuated EF-induced bradycardia.

### Acute effect of EF after nitric oxide synthase inhibition

**Fig. 5:**
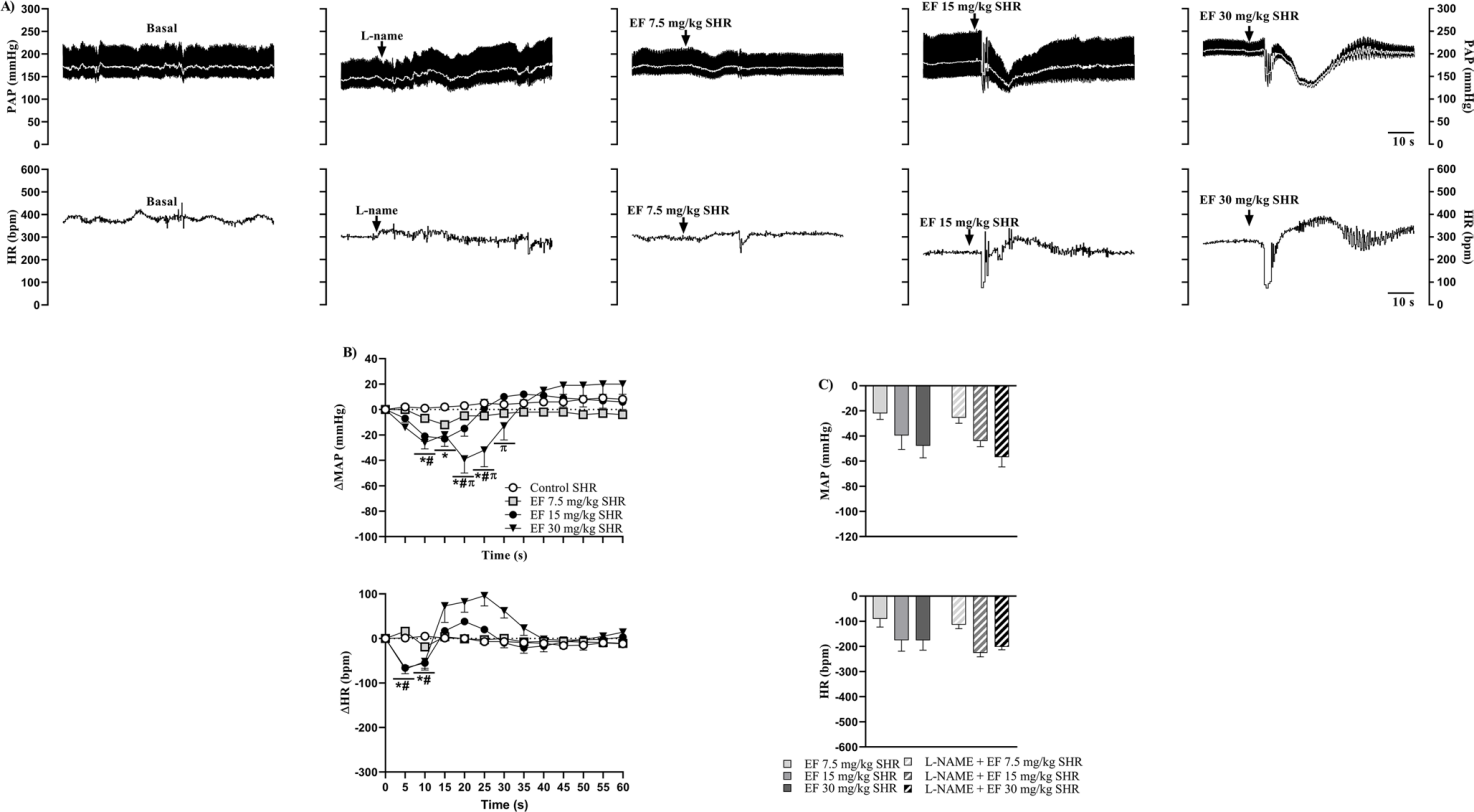
Representative recordings of PAP (upper panel, black trace), MAP (upper panel, white trace), and HR (lower panel) in SHR females (**A**) under basal conditions, with or without pre-treatment with L-NAME, followed by EF administration. Panel B shows changes in MAP (upper panel) and HR (lower panel) induced by EF, with or without L-NAME pre-treatment. Panel C shows maximum responses of MAP (upper panel) and HR (lower panel) induced by EF, with or without L-NAME pre-treatment. Data are expressed as mean ± SEM. *p < 0.05 vs. control; #*p* < 0.05 vs. 7.5 mg/kg; πp < 0.05 vs. 15 mg/kg

Figure 5 shows representative recordings of PAP (upper panel, black trace), MAP (upper panel, white trace), and HR (lower panel) in SHR females (A) under baseline conditions, after L-NAME administration, and subsequently treated with EF. Additionally, the effects of EF on MAP and HR over 60 s are presented for SHR females (B), along with maximal cardiovascular responses to EF in SHR females (C), pretreated or not with L-NAME.

Inhibition of nitric oxide synthase did not prevent the 15 and 30 mg/kg doses of EF from reducing MAP and HR compared with the control group. Cardiovascular parameters returned to baseline levels one minute after the last administration (Supplementary Tables 5 and Supplementary Fig. 5).

Analysis of maximal responses showed that L-NAME did not significantly attenuate reductions in MAP and HR relative to EF administered alone.

## Discussion

**Fig. 6:**
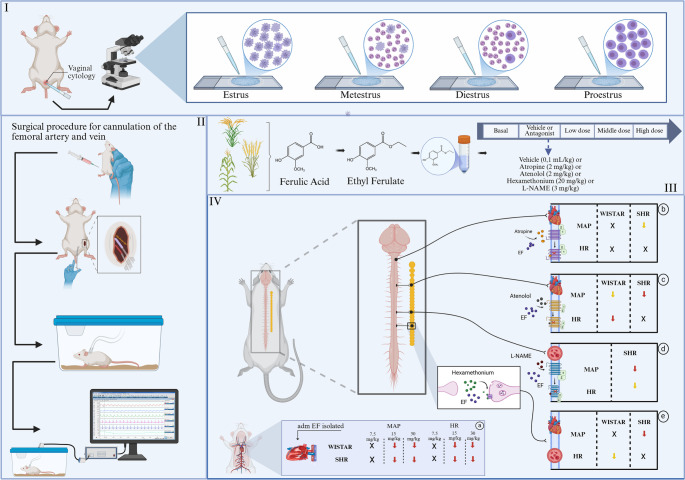
Graphical summary of the involvement of autonomic receptors in EF-induced hypotension and bradycardia in hypertensive female rats. (I) Determination of the estrous phase through vaginal cytology. (II) Cannulation of the femoral artery and vein 48 h prior to cardiovascular measurements. (III) Schematic representation of the experimental protocol for evaluation of cardiovascular parameters. (IV) Effects induced by EF administration alone (**a**), after atropine pre-treatment (**b**), after atenolol pre-treatment (**c**), after L-NAME pre-treatment (**d**), and after hexamethonium pre-treatment (**e**). Yellow arrows indicate partial reduction in blood pressure, red arrows indicate significant reduction, and black “x” indicates no change in blood pressure

In summary, this study aimed to evaluate the hypotensive and bradycardic effects of EF in female Wistar and SHR rats, as well as to elucidate the potential mechanisms underlying these effects. The results demonstrated that EF induced hypotension and bradycardia in both strains. Once the cardiovascular action of EF was confirmed, further investigation proceeded to identify the mechanisms responsible for these responses through selective autonomic receptor blockade and nitric oxide synthase inhibition, as illustrated in Fig. 6.

It is important to highlight that the majority of experimental studies are conducted in male rats, and clinical studies primarily involve hypertensive men, which limits the understanding of hypertension outcomes in female rats and women. This underscores the relevance of the present study, in which SHR females were used. Females in the metestrus and diestrus phases were specifically selected for the experiments, as hormone levels are more stable during these phases compared with other stages of the estrous cycle [24]. In this context, indirect estimation of sex hormone levels was performed through vaginal cytology 48 h prior to the experiment and immediately before cannulation, in order to avoid handling the females on the day of the experiment, which could induce stress and consequently affect data acquisition.

As expected and widely reported in the literature, 12-week-old SHR females exhibited elevated MAP levels, yet, surprisingly, baseline HR values were lower compared with Wistar females. Our data are consistent with previous studies; for instance, Maris and coworkers evaluated the influence of sex on MAP and HR in six-month-old Wistar Kyoto (WKY) and SHR rats and similarly observed that SHR females displayed elevated baseline MAP but reduced HR compared to controls [25]. These findings suggest that, under the experimental conditions described, SHR females already exhibit impaired cardiac electrical activity, which may be, at least in part, a consequence of cardiac hypertrophy, known to be present in SHR females from the 9th week of life [26, 27]. Furthermore, it was observed that in SHR, PAP is increased compared to normotensive females. These findings corroborate comparative studies between SHR and normotensive rats (Wistar and Wistar Kyoto), showing that the elevation in PAP levels present in SHR is associated with increased arterial stiffness and reduced compliance of large arteries, evidenced by the amplification of pulse wave velocity and reduced aortic distensibility because of endothelial dysfunction and molecular alterations in the vascular matrix [28–30].

As previously reported, only the 15 and 30 mg/kg doses of EF were able to significantly reduce MAP and HR in both Wistar and SHR females. To the best of our knowledge, there are no previous reports in the literature regarding the effects of EF on hypertension in SHR females.

On the other hand, ferulic acid, a precursor of EF, was evaluated by Suzuki and coworkers for its cardiovascular effects. Intravenous administration of ferulic acid (0.5 and 1.0 mg/kg) significantly reduced SAP in both Wistar and SHR rats, with SHR exhibiting a more pronounced decrease compared with Wistar rats [31].

Subsequently, another study evaluated a fraction of rice bran rich in ferulic acid, which was able to prevent blood pressure elevation in SHR rats predisposed to stroke. Notably, the compound also attenuated urinary concentrations of the oxidative stress marker 8-hydroxy-2’-deoxyguanosine (8-OHdG) [32].

After confirming that EF induces hypotension and bradycardia in Wistar and SHR females, the next step was to determine the potential mechanisms underlying these effects. In this context, the participation of autonomic pathways and nitric oxide in EF–induced cardiovascular responses were investigated.

Initially, muscarinic receptor blockade was performed through administration of atropine, a non-selective antagonist, to evaluate the involvement of the cholinergic pathway in the hemodynamic responses induced by EF. Thus, the results indicated that pretreatment with atropine completely prevented the reduction in cardiovascular parameters in Wistar females. In SHR females, muscarinic blockade prevented bradycardia but only partially attenuated the reduction in MAP. These findings suggest that the mechanism of action of EF in Wistar and SHR females involves M2 muscarinic receptors, since muscarinic receptors of the M2 subtype are predominantly located in the heart. Therefore, stimulation of M2 receptors influences the function of cardiac ion channels, promoting a decrease in inotropy and chronotropy through the modulation of different cellular pathways [33]. However, hypertensive female rats appear to possess at least one additional signaling pathway, since hypotension was not fully abolished after muscarinic blockade.

Next, we sought to evaluate the participation of cardiac adrenergic receptors in the EF-induced cardiovascular response. The results indicated that the cardiac adrenergic pathway is not involved in EF-induced hypotension in either strain, but it appears to contribute to bradycardia in SHR females. No studies in the literature were found that specifically assessed the role of β-adrenergic receptors in EF-induced cardiovascular response. However, it has been reported that hypotension induced by ferulic acid was not blocked by administration of propranolol, a non-selective β-adrenergic antagonist³¹.

Subsequently, ganglionic blockade was performed using hexamethonium, an antagonist of nicotinic receptors. Our results showed that the functional alterations induced by the development of hypertension modified the responsiveness of nicotinic receptors in SHR females, as hexamethonium only attenuated EF-induced bradycardia in SHR (only 15 mg/kg) and hypotension in Wistar females. Thus, in normotensive rats, the hypotensive effect of EF is, in part, dependent on autonomic ganglionic transmission, since pretreatment with hexamethonium attenuated the reduction in MAP. In contrast, in hypertensive females, the hypotensive effect was maintained even after ganglionic blockade, suggesting that evolution in hypertension was followed by loss of nicotinic receptor sensitivity to EF in SHR. This indicates that in normotensive rats, EF acts simultaneously on both nicotinic and muscarinic receptors. Notably, no previous studies have reported the effects of hexamethonium on EF- or ferulic acid–induced cardiovascular responses.

After evaluating the participation of major autonomic receptors in EF-induced hypotension and bradycardia, it became evident that, in SHR females, the blockade of the main autonomic pathways did not completely abolish the effects of EF. Therefore, the involvement of the nitric oxide (NO) pathway, which is widely recognized as a key vasodilatory mediator [33], was investigated. The results showed that NO did not alter EF-induced hypotension and bradycardia in SHR females. These findings are not consistent with those of Suzuki and colleagues, who reported that inhibition of NO synthesis by L-NAME suppressed ferulic acid-induced hypotension [31].

Moreover, caffeic acid, a phenylpropanoid similar to EF, has been shown to modulate the cardiovascular system. A previous study demonstrated that caffeic acid and its derivatives possess vasorelaxant activities, acting primarily on endothelial and vascular smooth muscle cells [34]. Taubert and partners observed that in pre-contracted porcine coronary arteries with PGF2α (prostaglandin F2α), caffeic acid induced NO release and vascular relaxation. This effect was abolished in endothelium-denuded vessels or after NOS inhibition with L-NMMA [35]. These in vitro findings further highlight the need to expand experimental approaches to investigate the effects of EF in the cardiovascular system, such as studies evaluating its effects on endothelial and vascular smooth muscle cells, considering that the vasorelaxant activity described for other phenylpropanoids may also be exhibited by EF.

Another point to consider is the ability of EF, as well as other ferulic acid derivatives, to cross the blood–brain barrier. Zhou and colleagues observed that administering different doses of ferulic acid methyl ester was able to reduce the area of cerebral infarction in rats with myocardial ischemia-reperfusion injury (CIRI), in addition to promoting an increase in Bcl-2 and VEGF content [36]. Similarly, Zou and colleagues demonstrated that intraperitoneal treatment with a pre-dissolved EF solution (15 mg/kg) administered 30 min, 24, and 48 h after transient middle cerebral artery occlusion (tMCAO) surgery to induce ischemic brain injury attenuated the neuroinflammation present in stroke, evidenced by reduced mRNA levels of IL-1β, IL-6, TNF-α, and iNOS [37]. Therefore, further studies are needed to evaluate the ability of EF to cross the blood–brain barrier and consequently exert central actions on the RVLM, a region critical for controlling vasomotor sympathetic tone [38]. These studies aim to assess the participation of possible central mechanisms in EF-induced hypotension and bradycardia.

Some limitations of this study should be acknowledged. Firstly, the experiments were performed under acute conditions, and thus the long-term cardiovascular and metabolic effects of EF remain to be determined. Second, only females in the metestrus and diestrus phases were evaluated to minimize hormonal variability, but this approach may limit the extrapolation of findings to other stages of the estrous cycle or to postmenopausal conditions. Third, although autonomic receptor blockade and nitric oxide synthase inhibition provided important mechanistic insights, additional signaling pathways, such as endothelial and vascular smooth muscle targets, were not investigated and may contribute to the cardiovascular actions of EF. Finally, as this study was conducted in rats, caution is warranted in extrapolating the results to humans, highlighting the need for further studies including chronic models, other sexes, and translational approaches.

## Conclusion

In summary, this study demonstrates that EF induces acute hypotension and bradycardia in both Wistar and SHR females, revealing distinct underlying mechanisms between normotensive and hypertensive animals. In normotensive females, the effects are mediated via muscarinic and nicotinic receptors, whereas in SHR females, hypotension is only partially dependent on muscarinic pathways, suggesting additional mechanisms in hypertension. These findings not only advance the understanding of EF cardiovascular actions in females --- a group underrepresented in preclinical research --- but also position EF as a promising candidate for novel antihypertensive therapies. Future studies should explore the potential central and vascular mechanisms underlying EF effects and evaluate its long-term therapeutic potential.

Baseline values of systolic blood pressure (SBP), diastolic blood pressure (DBP), mean arterial pressure (MAP), and heart rate (HR) in Wistar and SHR females. Data are expressed as mean ± SEM. *p < 0.001 vs. Wistar group.

## Supplementary Information

Below is the link to the electronic supplementary material.

Supplementary figure 7 Supplementary Material 1Changes in MAP (upper panel) and HR (lower panel) in Wistar (column A) and SHR (column B) females one minute after vehicle or EF administration. Data are expressed as mean ± SEM (PNG 553 KB)

High Resolution Image (TIF 4.68 MB)

Supplementary figure 8 Supplementary Material 2Changes in MAP (upper panel) and HR (lower panel) induced by EF in Wistar (column A) and SHR (column B) females one minute after pre-treatment with or without atropine. Data are expressed as mean ± SEM. (PNG 578 KB)

High Resolution Image (TIF 4.62 MB)

Supplementary figure 9 Supplementary Material 3Changes in MAP (upper panel) and HR (lower panel) induced by EF in Wistar (column A) and SHR (column B) females one minute after pre-treatment with or without atenolol. Data are expressed as mean ± SEM. (PNG 573 KB)

High Resolution Image (TIF 1.13 MB)

Supplementary figure 10 Supplementary Material 4Changes in MAP (upper panel) and HR (lower panel) induced by EF in Wistar (column A) and SHR (column B) females one minute after pre-treatment with or without hexamethonium. Data are expressed as mean ± SEM. (PNG 476 KB)

High Resolution Image (TIF 4.28 MB)

Supplementary figure 11 Supplementary Material 5Changes in MAP (upper panel) and HR (lower panel) induced by EF in SHR females one minute after pre-treatment with or without L-NAME. Data are expressed as mean ± SEM.(PNG 476 KB)

High Resolution Image (TIF 2.38 MB)

Supplementary Material 6 Changes in MAP and HR in Wistar and SHR females one minute after vehicle or EF administration. Data are expressed as mean ± SEM. (DOCX 1.97 MB)

Supplementary Material 7 Changes in MAP and HR induced by EF in Wistar and SHR females one minute after pre-treatment with or without atropine. Data are expressed as mean ± SEM.(DOCX 1.97 MB)

Supplementary Material 8 Changes in MAP and HR induced by EF in Wistar and SHR females one minute after pre-treatment with or without atenolol. Data are expressed as mean ± SEM.(DOCX 1.97 MB)

Supplementary Material 9 Changes in MAP and HR induced by EF in Wistar and SHR females one minute after pre-treatment with or without hexamethonium. Data are expressed as mean ± SEM.(DOCX 1.97 MB)

Supplementary Material 10 Changes in MAP and HR induced by EF in SHR females one minute after pre-treatment with or without L-NAME. Data are expressed as mean ± SEM.(DOCX 1.96 MB)

## Data Availability

No datasets were generated or analysed during the current study.
