# Supplementary figures and images for: Role of autonomic receptors in ethyl ferulate-induced cardiovascular effects in normotensive and hypertensive female rats
Source: Pflugers Arch. 2026 Apr 25;478(5):44. doi: 10.1007/s00424-026-03170-3 (PMC13110241; doi:10.1007/s00424-026-03170-3)

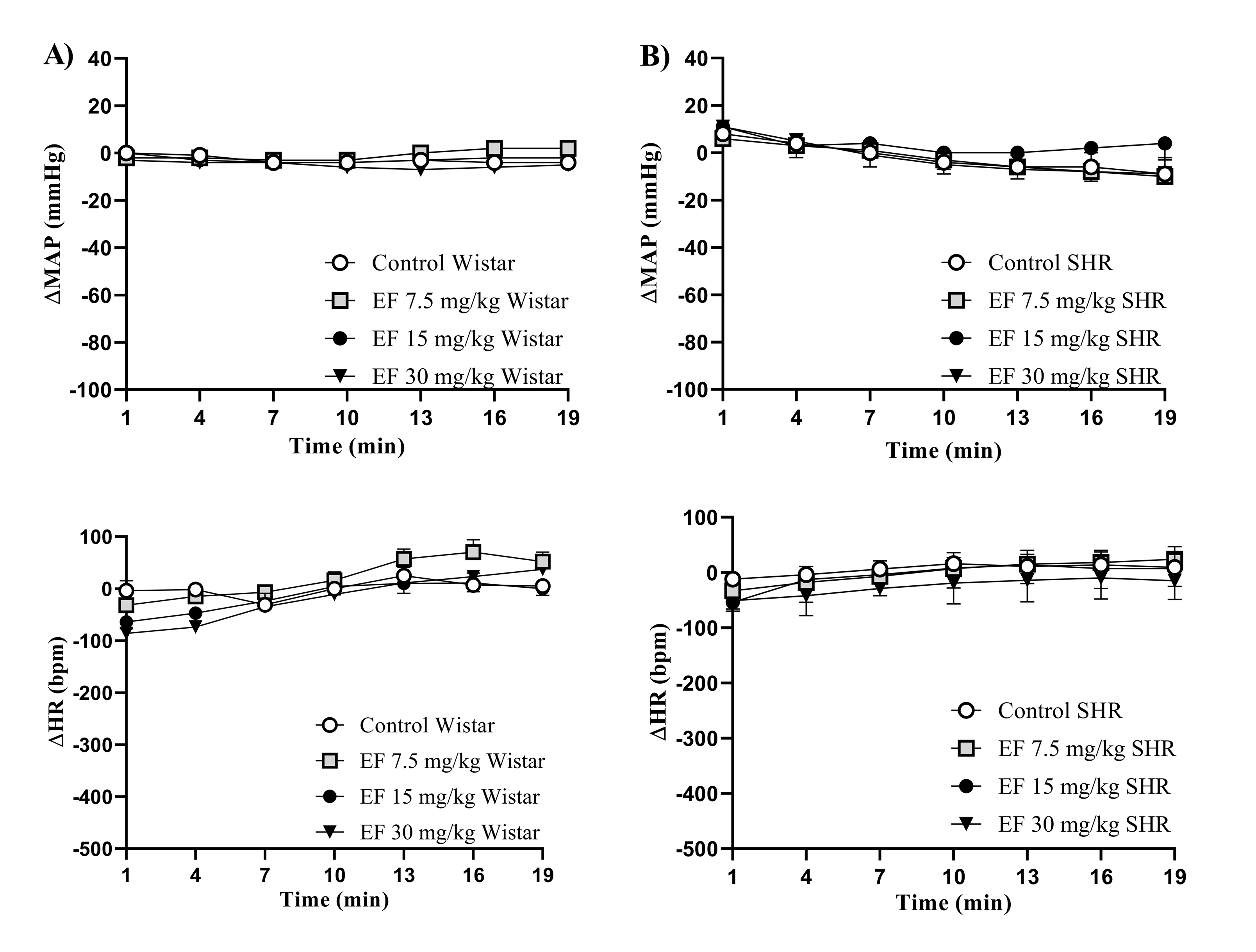

Supplement: Supplementary file 1 — Supplementary Material 1Changes in MAP (upper panel) and HR (lower panel) in Wistar (column A) and SHR (column B) females one minute after vehicle or EF administration. Data are expressed as mean ± SEM (PNG 553 KB) [file 424_2026_3170_Fig7_ESM.png]

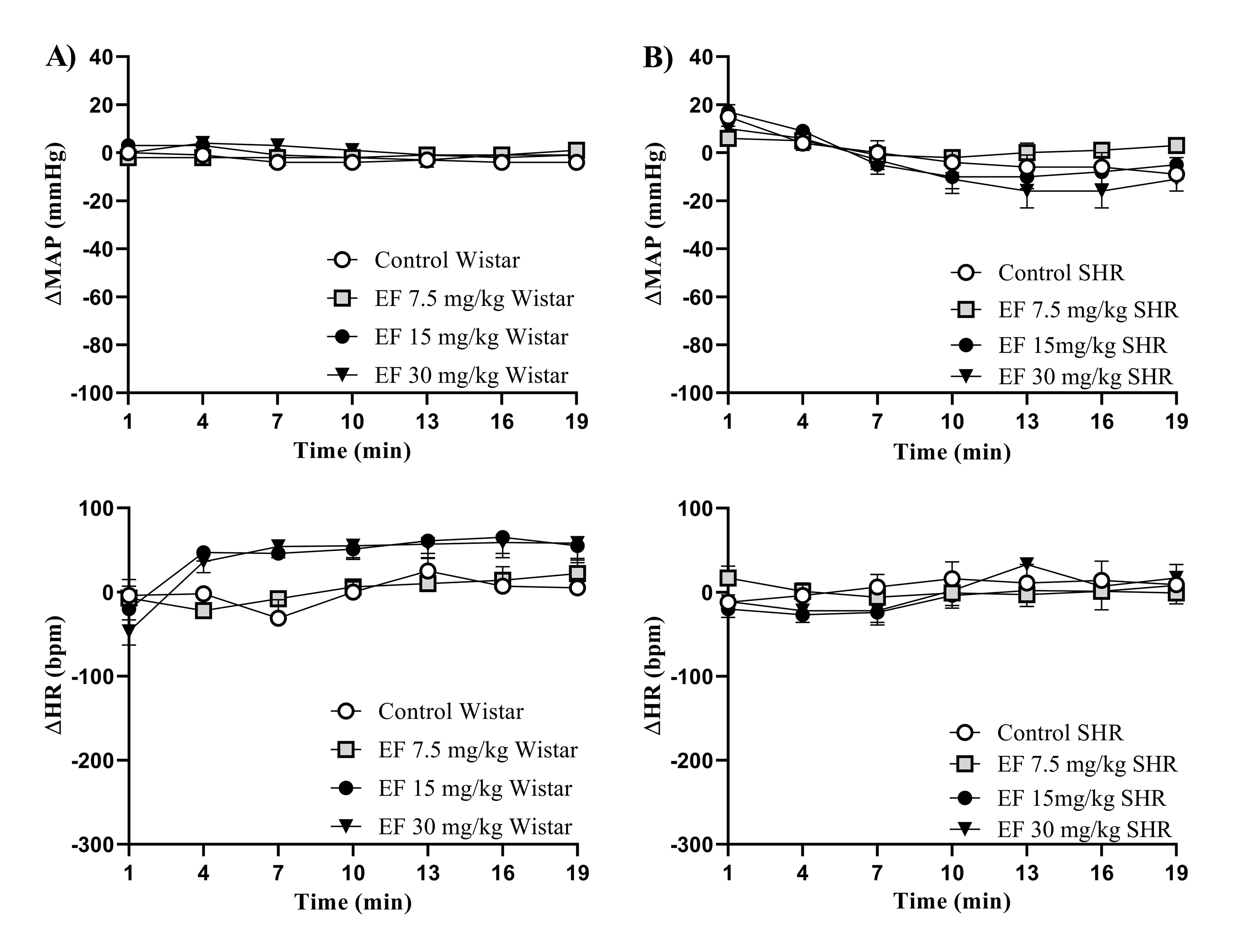

Supplement: Supplementary file 3 — Supplementary Material 2Changes in MAP (upper panel) and HR (lower panel) induced by EF in Wistar (column A) and SHR (column B) females one minute after pre-treatment with or without atropine. Data are expressed as mean ± SEM. (PNG 578 KB) [file 424_2026_3170_Fig8_ESM.png]

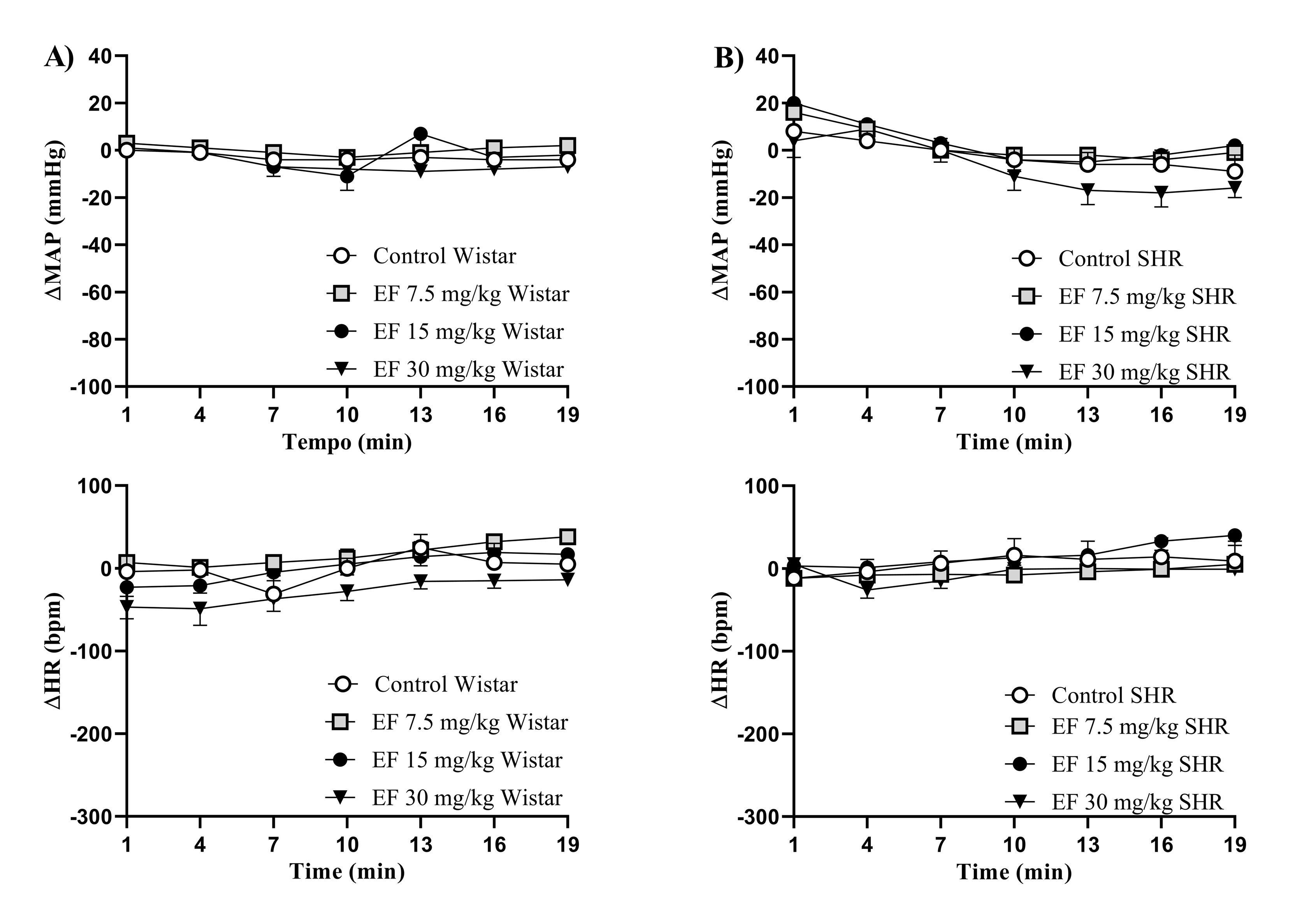

Supplement: Supplementary file 5 — Supplementary Material 3Changes in MAP (upper panel) and HR (lower panel) induced by EF in Wistar (column A) and SHR (column B) females one minute after pre-treatment with or without atenolol. Data are expressed as mean ± SEM. (PNG 573 KB) [file 424_2026_3170_Fig9_ESM.png]

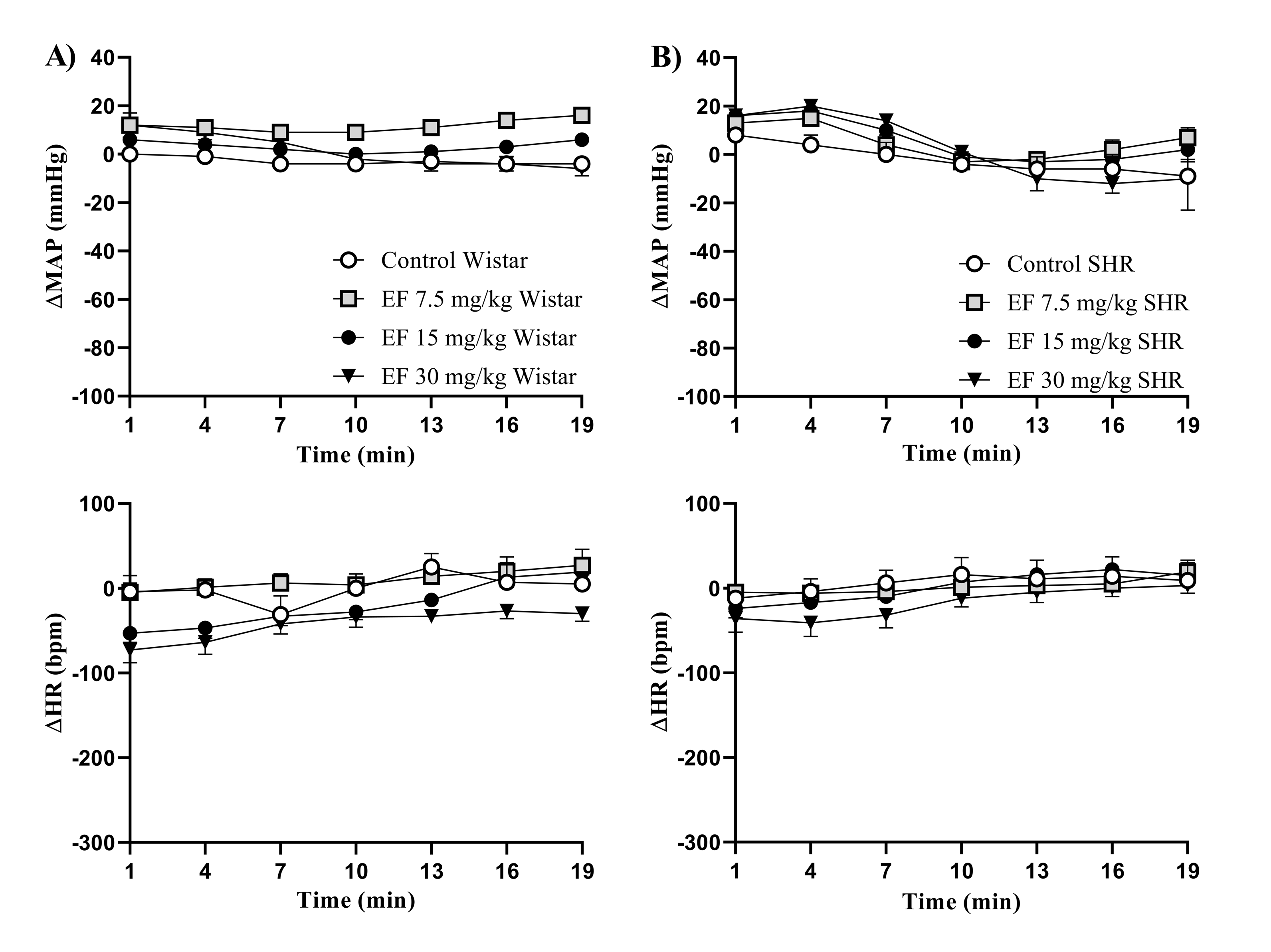

Supplement: Supplementary file 7 — Supplementary Material 4Changes in MAP (upper panel) and HR (lower panel) induced by EF in Wistar (column A) and SHR (column B) females one minute after pre-treatment with or without hexamethonium. Data are expressed as mean ± SEM. (PNG 476 KB) [file 424_2026_3170_Fig10_ESM.png]

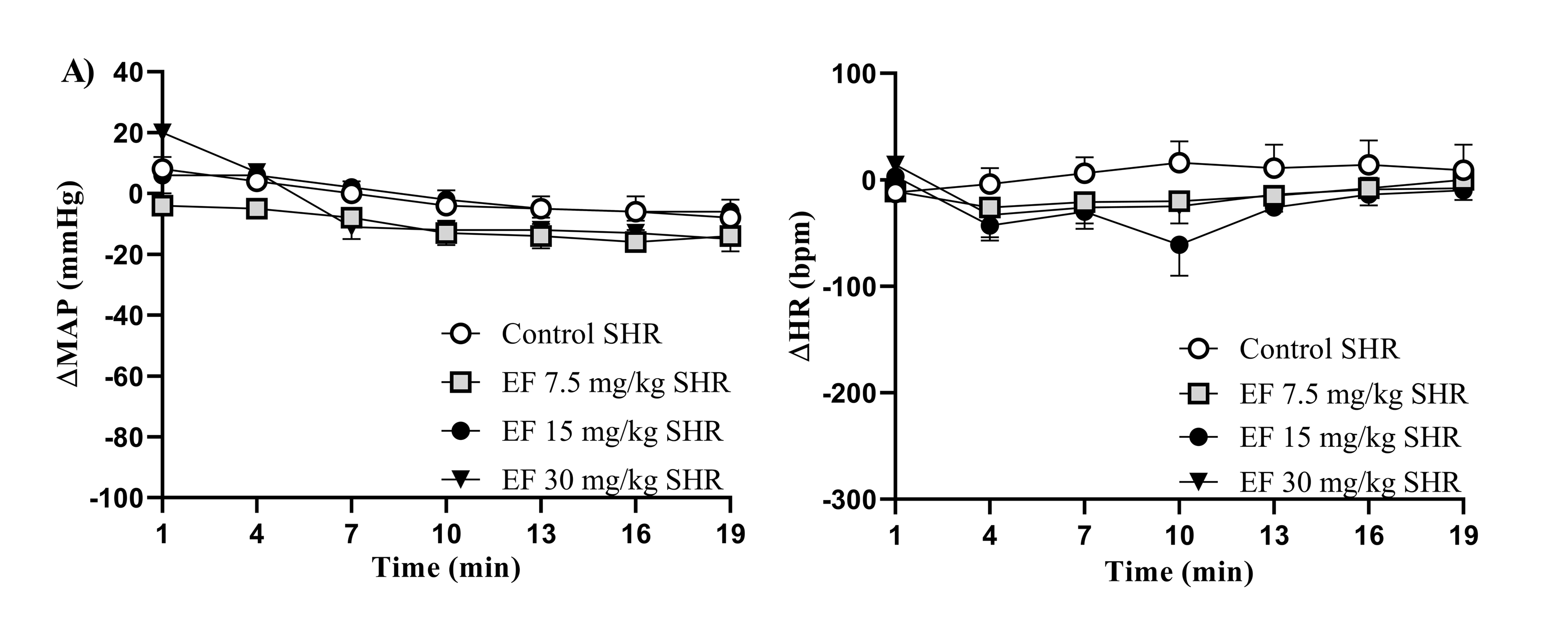

Supplement: Supplementary file 9 — Supplementary Material 5Changes in MAP (upper panel) and HR (lower panel) induced by EF in SHR females one minute after pre-treatment with or without L-NAME. Data are expressed as mean ± SEM.(PNG 476 KB) [file 424_2026_3170_Fig11_ESM.png]

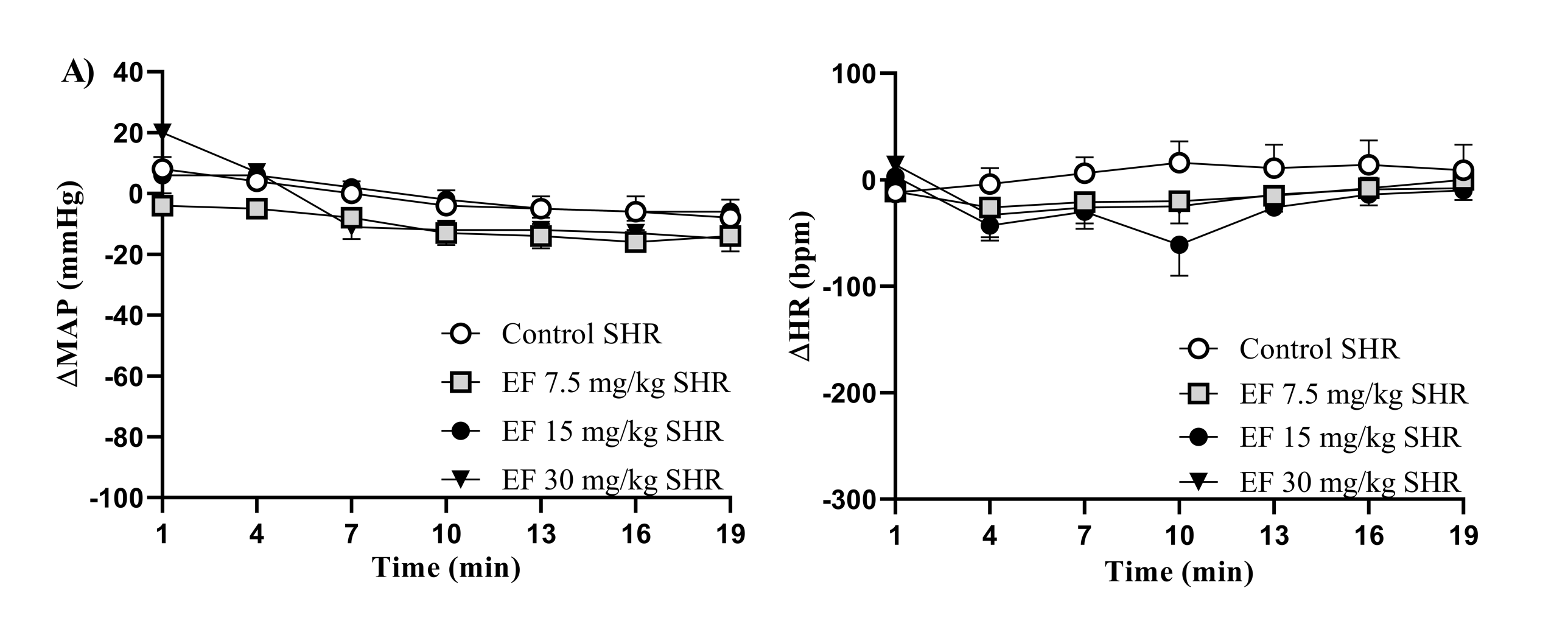

Supplement: Supplementary file 10 — High Resolution Image (TIF 2.38 MB) [file 424_2026_3170_MOESM5_ESM.tif]
