# Supplementary material for: Role of autonomic receptors in ethyl ferulate-induced cardiovascular effects in normotensive and hypertensive female rats
Source: Pflugers Arch. 2026 Apr 25;478(5):44. doi: 10.1007/s00424-026-03170-3 (PMC13110241; doi:10.1007/s00424-026-03170-3)
Supplement: Supplementary file 11 — Supplementary Material 6 Changes in MAP and HR in Wistar and SHR females one minute after vehicle or EF administration. Data are expressed as mean ± SEM. (DOCX 1.97 MB) [file 424_2026_3170_MOESM6_ESM.docx]

Supplementary Table 1

|  | **Control**  **Wistar** | | | **EF 7.5 mg/kg Wistar** | | | | **EF 15 mg/kg Wistar** | | | **EF 30 mg/kg Wistar** | | **Control**  **SHR** | | | **EF 7.5 mg/kg SHR** | | **EF 15 mg/kg SHR** | | **EF 30 mg/kg SHR** | |
| --- | --- | --- | --- | --- | --- | --- | --- | --- | --- | --- | --- | --- | --- | --- | --- | --- | --- | --- | --- | --- | --- |
| **Time**  **(s)** | **MAP**  **(mmHg)** | **HR (bpm)** | **MAP**  **(mmHg)** | | **HR (bpm)** | | **MAP**  **(mmHg)** | | **HR (bpm)** | **MAP**  **(mmHg)** | | **HR (bpm)** | **MAP**  **(mmHg)** | **HR (bpm)** | | **MAP (mmHg)** | **HR (bpm)** | **MAP**  **(mmHg)** | **HR (bpm)** | **MAP**  **(mmHg)** | **HR (bpm)** |
| **60-65** | 1 ± 3 | -25 ± 12 | | -1 ± 1 | | -24 ± 12 | | -3 ± 1 | -55 ± 18 | | -4 ± 1 | -89 ± 7 | 8 ± 4 | | 5 ± 11 | 5 ± 3 | -21 ± 19 | 9 ± 7 | -37 ± 24 | 16 ± 5 | -16 ± 24 |
| **65-70** | -1 ± 2 | -25 ± 12 | | -2 ± 1 | | -20 ± 14 | | -2 ± 1 | -55 ± 18 | | -5 ± 1 | -90 ± 10 | 8 ± 4 | | 3 ± 10 | 4 ± 4 | -16 ± 20 | 9 ± 7 | -37 ± 25 | 18 ± 5 | -20 ± 26 |
| **70-75** | 0 ± 2 | -19 ± 11 | | -2 ± 1 | | -18 ± 14 | | -3 ± 2 | -52 ± 17 | | -5 ± 1 | -92 ± 9 | 7 ± 4 | | 3 ± 11 | 5 ± 4 | -14 ± 19 | 9 ± 7 | -41 ± 27 | 18 ± 5 | -12 ± 26 |
| **75-80** | -1 ± 2 | -19 ± 10 | | -2 ± 1 | | -21 ± 14 | | -3 ± 2 | -46 ± 17 | | -5 ± 1 | -87 ± 6 | 7 ± 4 | | 4 ± 12 | 4 ± 4 | -14 ± 18 | 8 ± 7 | -34 ± 25 | 20 ± 5 | -16 ± 30 |
| **80-85** | -2 ± 2 | -15 ± 11 | | -2 ± 1 | | -22 ± 14 | | -3 ± 1 | -50 ± 17 | | -4 ± 1 | -85 ± 7 | 7 ± 4 | | 7 ± 16 | 6 ± 4 | -16 ± 18 | 6 ± 7 | -26 ± 26 | 18 ± 5 | -7 ± 34 |
| **85-90** | 0 ± 3 | -14 ± 10 | | -2 ± 1 | | -17 ± 14 | | -3 ± 2 | -53 ± 16 | | -4 ± 1 | -88 ± 10 | 8 ± 4 | | 4 ± 13 | 4 ± 4 | -10 ± 20 | 6 ± 7 | -23 ± 26 | 18 ± 5 | -2 ± 32 |
| **90-95** | -2 ± 2 | - 22 ± 12 | | -2 ± 1 | | -19 ± 13 | | -4 ± 1 | -51 ± 16 | | -4 ± 1 | -84 ± 9 | 8 ± 4 | | 3 ± 13 | 1 ± 4 | 10 ± 20 | 7 ± 7 | -25 ± 27 | 17 ± 6 | -12 ± 30 |
| **95-100** | -1 ± 3 | -23 ± 10 | | -3 ± 1 | | -20 ± 11 | | -3 ± 1 | -48 ± 15 | | -4 ± 1 | -75 ± 9 | 9 ± 4 | | 2 ± 14 | 0 ± 5 | -14 ± 18 | 6 ± 7 | -26 ± 27 | 18 ± 5 | -3 ± 32 |
| **100-105** | -1 ± 2 | -21 ± 10 | | -3 ± 1 | | -22 ± 12 | | -3 ± 1 | -48 ± 16 | | -2 ±1 | -71 ± 9 | 8 ± 4 | | 2 ± 12 | 1 ± 5 | -21 ± 17 | 5 ± 7 | -28 ± 30 | 17 ± 5 | 3 ± 34 |
| **105-110** | -2 ± 2 | -23 ± 12 | | -3 ± 1 | | -17 ± 12 | | -3 ± 1 | -50 ± 15 | | -3 ± 1 | -67 ± 10 | 8 ± 4 | | 4 ± 12 | 0 ± 5 | -20 ± 16 | 3 ± 6 | -29 ± 31 | 15 ± 4 | -1 ± 34 |
| **110-115** | -1 ± 3 | -24 ± 11 | | -3 ± 1 | | -17 ± 13 | | -3 ± 1 | -57 ± 15 | | -3 ± 1 | -71 ± 12 | 7 ± 4 | | 7 ± 12 | 2 ± 4 | -15 ± 15 | 3 ± 6 | -26 ± 30 | 15 ± 4 | -3 ± 34 |
| **115-120** | -2 ± 2 | -18 ± 12 | | -3 ± 1 | | -15 ± 14 | | -3 ± 1 | -57 ± 16 | | -3 ± 1 | -74 ± 12 | 9 ± 4 | | 4 ± 11 | 2 ± 5 | -20 ± 15 | 2 ± 6 | -22 ± 32 | 13 ± 4 | -2 ± 35 |
| **120-125** | -1 ± 3 | -20 ± 13 | | -2 ± 1 | | -16 ± 12 | | -3 ± 1 | -53 ± 15 | | -2 ± 2 | -70 ± 13 | 4 ± 4 | | 5 ± 11 | 2 ± 5 | -23 ± 16 | 3 ± 6 | -25 ± 34 | 12 ± 5 | 7 ± 34 |
| **125-130** | -1 ± 3 | -17 ± 12 | | -2 ± 1 | | -14 ± 11 | | -3 ± 1 | -49 ± 17 | | -3 ± 1 | -70 ± 13 | 3 ± 4 | | 13 ± 12 | 2 ± 4 | -15 ± 14 | 2 ± 6 | -17 ± 32 | 12 ± 5 | 3 ± 34 |
| **130-135** | -3 ± 3 | -12 ± 13 | | -2 ± 1 | | -14 ± 12 | | -3 ± 1 | -53 ± 15 | | -3 ± 1 | -69 ± 13 | 5 ± 4 | | 11 ± 12 | 4 ± 5 | -14 ± 15 | 1 ± 5 | -12 ± 31 | 12 ± 5 | 3 ± 34 |
| **135-140** | -2 ± 2 | -9 ± 14 | | -3 ± 1 | | -20 ± 13 | | -3 ± 1 | -47 ± 14 | | -3 ± 1 | -67 ± 13 | 7 ± 4 | | 11 ± 10 | 2 ± 5 | -10 ± 18 | 0 ± 6 | -15 ± 30 | 12 ± 5 | 5 ± 34 |
| **140-145** | -1 ± 2 | -4 ± 14 | | -3 ± 1 | | -22 ± 12 | | -3 ± 1 | -46 ± 15 | | -4 ± 1 | -67 ± 12 | 4 ± 4 | | 9 ± 13 | 4 ± 4 | -12 ± 18 | 1 ± 6 | -14 ± 31 | 10 ± 5 | 0 ± 32 |
| **145-150** | -2 ± 2 | -7 ± 13 | | -2 ± 1 | | -18 ± 12 | | -3 ± 1 | -47 ± 15 | | -3 ± 1 | -64 ± 12 | 6 ± 3 | | 6 ± 12 | 4 ± 4 | -13 ± 16 | 1 ± 6 | -18 ± 32 | 10 ± 5 | 3 ± 34 |
| **150-155** | -1 ± 2 | -22 ± 19 | | -2 ± 1 | | -13 ± 11 | | -3 ± 1 | -50 ± 15 | | -3 ± 1 | -63 ± 9 | 1 ± 4 | | 9 ± 11 | 3 ± 4 | -18 ± 17 | 1 ± 7 | -13 ± 33 | 11 ± 5 | 11 ± 35 |
| **155-160** | -1 ± 3 | -29 ± 21 | | -2 ± 1 | | -14 ± 11 | | -4 ± 1 | -49 ± 15 | | -3 ± 2 | -61 ± 11 | -1 ± 5 | | 10 ± 11 | 4 ± 4 | -17 ± 16 | 3 ± 6 | -12 ± 34 | 9 ± 5 | 13 ± 37 |
| **160-165** | -2 ± 2 | -15 ± 14 | | -2 ± 1 | | -16 ± 11 | | -4 ± 1 | -47 ± 15 | | -3 ± 1 | -63 ± 10 | 2 ± 3 | | 7 ± 11 | 3 ± 4 | -22 ± 17 | 2 ± 6 | -20 ± 36 | 10 ± 5 | 13 ± 36 |
| **165-170** | -3 ± 2 | -18 ± 13 | | -2 ± 1 | | -15 ± 12 | | -2 ± 1 | -40 ± 15 | | -3 ± 1 | -62 ± 11 | 4 ± 3 | | 4 ± 11 | 3 ± 4 | -25 ± 18 | 2 ± 6 | -21 ± 34 | 9 ± 5 | 18 ± 39 |
| **170-175** | -2 ± 2 | -14 ± 11 | | -2 ± 1 | | -21 ± 11 | | -3 ± 1 | -41 ± 15 | | -3 ± 1 | -64 ± 12 | 3 ± 4 | | 3 ± 11 | 4 ± 4 | -21 ± 18 | 3 ± 7 | -24 ± 34 | 8 ± 5 | 16 ± 38 |
| **175-180** | -2 ± 2 | -11 ± 12 | | -2 ± 1 | | -15 ± 9 | | -3 ± 1 | -46 ± 15 | | -3 ± 1 | -64 ± 13 | 2 ± 4 | | 3 ± 11 | 4 ± 4 | -26 ± 19 | 3 ± 6 | -22 ± 34 | 5 ± 6 | 13 ± 38 |
| **180-185** | -3 ± 2 | -17 ± 13 | | -2 ± 1 | | -14 ± 8 | | -4 ± 1 | -50 ± 15 | | -3 ± 1 | -63 ± 13 | 3 ± 4 | | 4 ± 10 | 4 ± 5 | -23 ± 18 | 2 ± 6 | -17 ± 34 | 6 ± 6 | 15 ± 39 |
| **185-190** | -3 ± 2 | -17 ± 12 | | -2 ± 1 | | -18 ± 10 | | -3 ± 1 | -46 ± 15 | | -4 ± 1 | -62 ± 13 | 2 ± 4 | | 1 ± 10 | 3 ± 4 | -24 ± 17 | 2 ± 6 | -18 ± 34 | 6 ± 5 | 15 ± 40 |
| **190-195** | -3 ± 2 | -21 ± 12 | | -2 ± 1 | | -14 ± 10 | | -2 ± 2 | -39 ± 14 | | -4 ± 1 | -58 ± 14 | 2 ± 4 | | -1 ± 12 | 3 ± 4 | -22 ± 16 | 3 ± 6 | -17 ± 33 | 6 ± 5 | 13 ± 39 |
| **195-200** | -3 ± 2 | -20 ± 11 | | -3 ± 1 | | -17 ± 12 | | -3 ± 2 | -33 ± 12 | | -3 ± 1 | -58 ± 15 | 1 ± 3 | | 0 ± 11 | 2 ± 4 | -23 ± 17 | 3 ± 6 | -20 ± 33 | 6 ± 5 | 12 ± 39 |
| **200-205** | -2 ± 2 | -20 ± 10 | | -2 ± 2 | | -14 ± 15 | | -2 ± 1 | -31 ± 13 | | -3 ± 1 | -54 ± 14 | 2 ± 4 | | -1 ± 12 | 2 ± 4 | -23 ± 17 | 2 ± 6 | -17 ± 35 | 6 ± 5 | -9 ± 39 |
| **205-210** | -2 ± 2 | -18 ± 10 | | -1 ± 2 | | -12 ± 14 | | -3 ± 1 | -37 ± 15 | | -3 ± 1 | -55 ± 15 | 0 ± 4 | | 2 ± 11 | 2 ± 4 | -23 ± 19 | 4 ± 6 | -16 ± 35 | 6 ± 4 | 13 ± 38 |
| **210-215** | -3 ± 1 | -18 ± 9 | | -2 ± 1 | | 0 ± 20 | | -3 ± 1 | -37 ± 15 | | -2 ± 1 | -55 ± 14 | -1 ± 3 | | 7 ± 11 | 0 ± 4 | -18 ± 18 | 2 ± 6 | -14 ± 34 | 6 ± 5 | 11 ± 37 |
| **215-220** | -2 ± 2 | -15 ± 7 | | -2 ± 1 | | -2 ± 17 | | -2 ± 2 | -30 ± 15 | | -4 ± 1 | -53 ± 13 | -3 ± 3 | | 9 ± 12 | 3 ± 4 | -17 ± 17 | 0 ± 5 | -17 ± 36 | 6 ± 5 | 11 ± 35 |
| **220-225** | -3 ± 1 | -15 ± 7 | | -2 ± 2 | | -8 ± 17 | | -4 ± 2 | -37 ± 15 | | -3 ± 1 | -50 ± 14 | 1 ± 4 | | 7 ± 12 | 2 ± 5 | -14 ± 18 | 0 ± 6 | -15 ± 36 | 6 ± 5 | 10 ± 36 |
| **225-230** | -3 ± 1 | -18 ± 9 | | -2 ± 1 | | -5 ± 15 | | -3 ± 1 | -37 ± 14 | | -3 ± 1 | -50 ± 13 | 0 ± 3 | | 4 ± 11 | 3 ± 5 | -10 ± 20 | 1 ± 7 | -17 ± 37 | 5 ± 5 | 9 ± 35 |
| **230-235** | -3 ± 2 | -17 ± 10 | | -2 ± 1 | | -7 ± 18 | | -3 ± 1 | -35 ± 15 | | -4 ± 1 | -46 ± 13 | 1 ± 4 | | 2 ± 12 | 3 ± 4 | -8 ± 21 | 2 ± 6 | -18 ± 38 | 6 ± 4 | 7 ± 36 |
| **235-240** | -2 ± 1 | -14 ± 9 | | -1 ± 2 | | -9 ± 17 | | -4 ± 2 | -39 ± 15 | | -4 ± 1 | -45 ± 13 | 1 ± 4 | | 2 ± 10 | 3 ± 4 | -11 ± 18 | 2 ± 5 | -11 ± 35 | 6 ± 5 | 8 ± 36 |
| **240-245** | -2 ± 1 | -1 ± 9 | | -3 ± 2 | | -9 ± 16 | | -5 ± 2 | -40 ± 15 | | -5 ± 1 | -47 ± 13 | 0 ± 4 | | 1 ± 12 | 4 ± 4 | -11 ± 16 | 0 ± 6 | -19 ± 40 | 6 ± 4 | 9 ± 36 |
| **245-250** | -3 ± 1 | -7 ± 9 | | -2 ± 1 | | -4 ± 18 | | -4 ± 2 | -39 ± 15 | | -4 ± 1 | -48 ± 14 | 1 ± 2 | | 7 ± 17 | 4 ± 5 | -8 ± 20 | -2 ± 4 | -18 ± 39 | 6 ± 5 | 9 ± 37 |
| **250-255** | -3 ± 1 | -8 ± 7 | | -2 ± 1 | | -6 ± 16 | | -4 ± 1 | -37 ± 14 | | -4 ± 1 | -46 ± 14 | 0 ± 2 | | 8 ± 19 | 1 ± 4 | -12 ± 18 | -4 ± 5 | -13 ± 39 | 6 ± 4 | 9 ± 36 |
| **255-260** | -3 ± 1 | -2 ± 9 | | -2 ± 1 | | -8 ± 15 | | -4 ± 1 | -28 ± 15 | | -4 ± 2 | -47 ± 15 | 3 ± 2 | | 6 ± 17 | 3 ± 3 | -13 ± 18 | -2 ± 5 | -9 ± 39 | 6 ± 4 | 9 ± 36 |
| **260-265** | -3 ± 1 | 0 ± 10 | | -3 ± 1 | | -12 ± 15 | | -4 ± 2 | -26 ± 15 | | -4 ± 1 | -46 ± 15 | 2 ± 2 | | 4 ± 16 | 1 ± 4 | -13 ± 17 | -1 ± 6 | -11 ± 40 | 5 ± 4 | 10 ± 36 |
| **265-270** | -3 ± 1 | -1 ± 10 | | -2 ± 1 | | -7 ± 16 | | -4 ± 1 | -35 ± 14 | | -4 ± 1 | -45 ± 15 | 3 ± 2 | | 7 ± 17 | 0 ± 3 | -14 ± 18 | 1 ± 6 | -13 ± 40 | 5 ± 4 | 12 ± 36 |
| **270-275** | -3 ± 1 | 2 ± 10 | | -2 ± 1 | | -8 ± 14 | | -4 ± 2 | -30 ± 14 | | -4 ± 1 | -43 ± 14 | 1 ± 2 | | 12 ± 21 | -1 ± 3 | -11 ± 26 | 1 ± 6 | -13 ± 39 | 5 ± 4 | 12 ± 36 |
| **275-280** | -3 ± 1 | -3 ± 8 | | -2 ± 1 | | -12 ± 13 | | -4 ± 2 | -28 ± 14 | | -4 ± 1 | -39 ± 14 | 3 ± 2 | | 13 ± 23 | 0 ± 4 | -14 ± 20 | 2 ± 7 | -11 ± 40 | 5 ± 4 | 19 ± 33 |
| **280-285** | -3 ± 1 | -1 ± 10 | | -3 ± 1 | | -13 ± 14 | | -4 ± 1 | -29 ± 14 | | -4 ± 1 | -39 ± 15 | 3 ± 2 | | 11 ± 23 | 0 ± 4 | -14 ± 18 | 2 ± 7 | -15 ± 40 | 6 ± 4 | 14 ± 33 |
| **285-290** | -3 ± 1 | -9 ± 8 | | -3 ± 1 | | -15 ± 14 | | -4 ± 1 | -23 ± 15 | | -4 ± 1 | -44 ± 14 | 1 ± 3 | | 13 ± 25 | 1 ± 4 | -14 ± 17 | 2 ± 6 | -13 ± 39 | 5 ± 4 | 12 ± 34 |
| **290-295** | -2 ± 1 | -5 ± 15 | | -3 ± 1 | | -14 ± 15 | | -4 ± 1 | -22 ± 15 | | -4 ± 1 | -40 ± 14 | 2 ± 2 | | 13 ± 24 | 0 ± 4 | -13 ± 17 | 2 ± 6 | -18 ± 41 | 6 ± 6 | 13 ± 32 |
| **295-300** | -4 ± 1 | -8 ± 9 | | -2 ± 1 | | -4 ± 13 | | -4 ± 2 | -29 ± 15 | | -4 ± 1 | -35 ± 14 | 2 ± 3 | | 10 ± 21 | 0 ± 4 | 9 ± 17 | 1 ± 7 | -10 ± 39 | 4 ± 4 | 10 ± 34 |
|  |  |  | |  | |  | |  |  | |  |  |  | |  |  |  |  |  |  |  |
