# Supplementary material for: Role of autonomic receptors in ethyl ferulate-induced cardiovascular effects in normotensive and hypertensive female rats
Source: Pflugers Arch. 2026 Apr 25;478(5):44. doi: 10.1007/s00424-026-03170-3 (PMC13110241; doi:10.1007/s00424-026-03170-3)
Supplement: Supplementary file 12 — Supplementary Material 7 Changes in MAP and HR induced by EF in Wistar and SHR females one minute after pre-treatment with or without atropine. Data are expressed as mean ± SEM.(DOCX 1.97 MB) [file 424_2026_3170_MOESM7_ESM.docx]

Supplementary Table 2

|  | **Atropine**  **EF 7.5 mg/kg Wistar** | | **Atropine**  **EF 15 mg/kg Wistar** | | **Atropine**  **EF 30 mg/kg Wistar** | | **Atropine**  **EF 7.5 mg/kg SHR** | | **Atropine**  **EF 15 mg/kg**  **SHR** | | **Atropine**  **EF 30 mg/kg**  **SHR** | |
| --- | --- | --- | --- | --- | --- | --- | --- | --- | --- | --- | --- | --- |
| **Time (s)** | **MAP**  **(mmHg)** | **HR**  **(bpm)** | **MAP**  **(mmHg)** | **HR**  **(bpm)** | **MAP**  **(mmHg)** | **HR**  **(bpm)** | **MAP**  **(mmHg)** | **HR**  **(bpm)** | **MAP**  **(mmHg)** | **HR**  **(bpm)** | **MAP**  **(mmHg)** | **HR**  **(bpm)** |
| **60-65** | 0 ± 1 | -24 ± 9 | 3 ± 2 | -18 ± 14 | 0 ± 0 | -49 ± 14 | 10 ± 2 | 17 ± 13 | 13 ± 7 | 22 ± 10 | 13 ± 6 | -8 ± 21 |
| **65-70** | -1 ± 1 | -24 ± 8 | 4 ± 2 | -15 ± 15 | 0 ± 0 | -49 ± 14 | 8 ± 2 | 16 ±13 | 12 ± 7 | -22 ± 9 | 13 ± 6 | -7 ± 22 |
| **70-75** | -1 ± 1 | -23 ± 8 | 6 ± 3 | -14 ± 16 | 0 ± 1 | -49 ± 14 | 8 ± 1 | 14 ± 11 | 12 ± 7 | -24 ± 10 | 13 ± 7 | -7 ± 22 |
| **75-80** | -1 ± 1 | -26 ± 8 | 6 ± 3 | -15 ± 16 | 0 ± 1 | -49 ± 14 | 8 ± 2 | 13 ± 12 | 12 ± 6 | -25 ± 11 | 12 ± 7 | -8 ± 22 |
| **80-85** | -2 ± 1 | -26 ± 8 | 3 ± 2 | -17 ± 15 | 1 ± 0 | -48 ± 15 | 8 ± 2 | 12 ± 12 | 10 ± 6 | -23 ± 11 | 12 ± 6 | -9 ± 22 |
| **85-90** | -1 ± 1 | -26 ± 7 | 2 ± 2 | -16 ± 14 | 1 ± 1 | -50 ± 14 | 8 ± 2 | 8 ± 10 | 10 ± 5 | -22 ± 11 | 11 ± 5 | -11 ± 22 |
| **90-95** | -2 ± 1 | -24 ± 9 | 1 ± 2 | -15 ± 15 | 1 ± 1 | -49 ± 14 | 7 ± 1 | 7 ± 9 | 8 ± 4 | -24 ± 11 | 10 ± 5 | -12 ± 23 |
| **95-100** | -1 ± 1 | -23 ± 8 | 3 ± 1 | -17 ± 15 | 1 ± 0 | -48 ± 14 | 6 ± 1 | 7 ± 8 | 0 ± 5 | -24 ± 12 | 11 ± 5 | -12 ± 22 |
| **100-105** | -1 ± 1 | -24 ± 8 | 2 ± 2 | -17 ± 15 | 1 ± 1 | -47 ± 14 | 6 ± 2 | 5 ± 8 | 9 ± 5 | -23 ± 13 | 11 ± 5 | -14 ± 22 |
| **105-110** | -1 ± 1 | -25 ± 7 | 1 ± 1 | -16 ± 14 | 2 ± 1 | -46 ± 14 | 5 ± 1 | -2 ± 1 | 3 ± 5 | -24 ± 13 | 11 ± 5 | -17 ± 21 |
| **110-115** | -1 ± 1 | -24 ± 8 | 1 ± 1 | -12 ± 13 | 2 ± 1 | -46 ± 14 | 5 ± 2 | 2 ± 7 | 9 ± 5 | -24 ± 13 | 10 ± 4 | -17 ± 21 |
| **115-120** | -1 ± 1 | -21 ± 9 | 3 ± 1 | -12 ± 13 | 1 ± 1 | -46 ± 14 | 5 ± 2 | 2 ± 7 | 5 ± 7 | -23 ± 13 | 8 ± 4 | -18 ± 20 |
| **120-125** | -2 ± 1 | -24 ± 7 | 2 ± 1 | -14 ± 13 | 2 ± 1 | -47 ± 12 | 6 ± 2 | 0 ± 7 | 4 ± 6 | -20 ± 13 | 8 ± 5 | -19 ± 21 |
| **125-130** | -2 ± 1 | -22 ± 6 | 2 ± 1 | -13 ± 13 | 1 ± 1 | -46 ± 13 | 5 ± 3 | -2 ± 7 | 5 ± 5 | -20 ± 14 | 7 ± 4 | -21 ± 21 |
| **130-135** | -2 ± 1 | -22 ± 6 | 2 ± 1 | -14 ± 13 | 2 ± 1 | -44 ± 13 | 6 ± 3 | 0 ± 6 | 5 ± 4 | -25 ± 12 | 5 ± 4 | -21 ± 21 |
| **135-140** | -2 ± 1 | -2 ± 21 | 2 ± 1 | -14 ± 13 | 1 ± 1 | -39 ± 16 | 5 ± 2 | -1 ± 7 | 7 ± 4 | -26 ± 11 | 5 ± 4 | -23 ± 20 |
| **140-145** | -1 ± 0 | -4 ± 16 | 2 ± 1 | -15 ± 13 | 2 ± 0 | -41 ± 14 | 4 ± 2 | -3 ± 6 | 7 ± 4 | -29 ± 11 | 5 ± 3 | -23 ± 18 |
| **145-150** | -1 ± 1 | -8 ± 12 | 3 ± 1 | -15 ± 12 | 2 ± 1 | -44 ± 13 | 3 ± 2 | -2 ± 7 | 7 ± 3 | -30 ± 11 | 4 ± 3 | -22 ± 18 |
| **150-155** | 0 ± 1 | -19 ± 6 | 2 ± 2 | -15 ± 11 | 2 ± 0 | -46 ± 13 | 2 ± 2 | -3 ± 7 | 8 ± 3 | -31 ± 11 | 4 ± 3 | -23 ± 18 |
| **155-160** | -1 ± 1 | -17 ± 7 | 1 ± 1 | -15 ± 12 | 1 ± 1 | -45 ± 13 | 4 ± 2 | -3 ± 8 | 8 ± 3 | -31 ± 12 | 4 ± 3 | -24 ± 18 |
| **160-165** | -1 ± 1 | -16 ± 7 | 2 ± 2 | -16 ± 11 | 2 ± 1 | -44 ± 13 | 5 ± 2 | -3 ± 7 | 7 ± 3 | -32 ± 12 | 3 ± 3 | -24 ± 19 |
| **165-170** | -1 ± 1 | -18 ± 7 | 2 ± 2 | -17 ± 11 | 1 ± 0 | -45 ± 13 | 5 ± 2 | -3 ± 3 | 7 ± 3 | -33 ± 12 | 4 ± 3 | -23 ± 20 |
| **170-175** | -2 ± 1 | -18 ± 6 | 2 ± 1 | -17 ± 11 | 1 ± 1 | -46 ± 12 | 4 ± 2 | -3 ± 7 | 7 ± 3 | -30 ± 11 | 4 ± 3 | -26 ± 20 |
| **175-180** | -1 ± 1 | -17 ± 5 | 1 ± 1 | -14 ± 10 | 0 ± 1 | -46 ± 13 | 4 ± 1 | -3 ± 8 | 7 ± 2 | -28 ± 10 | 3 ± 3 | -26 ± 20 |
| **180-185** | -2 ± 1 | -18 ± 6 | 3 ± 2 | -15 ± 9 | 1 ± 1 | -41 ± 13 | 3 ± 1 | -4 ± 7 | 8 ± 2 | -25 ± 9 | 3 ± 3 | -25 ± 21 |
| **185-190** | -2 ± 1 | -17 ± 7 | 2 ± 1 | -17 ± 8 | 1 ± 1 | -43 ± 13 | 4 ± 2 | -7 ± 7 | 7 ± 2 | -26 ± 9 | 2 ± 4 | -24 ± 21 |
| **190-195** | -2 ± 0 | -19 ± 6 | 2 ± 1 | -16 ± 9 | 1 ± 1 | -43 ± 13 | 3 ± 1 | -7 ± 7 | 7 ± 2 | -25 ± 7 | 2 ± 3 | -24 ± 21 |
| **195-200** | -2 ± 1 | -15 ± 6 | 2 ± 2 | -16 ± 9 | 1 ± 1 | -42 ± 13 | 3 ± 2 | -8 ± 6 | 6 ± 2 | -25 ± 7 | 2 ± 4 | -27 ± 19 |
| **200-205** | -3 ± 1 | -15 ± 6 | 2 ± 2 | -17 ± 9 | 1 ± 1 | -42 ± 13 | 3 ± 2 | -9 ± 6 | 3 ± 5 | -21 ± 12 | 3 ± 4 | -29 ± 18 |
| **205-210** | -2 ± 1 | -12 ± 7 | 1 ± 2 | -15 ± 8 | 1 ± 1 | -41 ± 13 | 3 ± 2 | -9 ± 6 | 3 ± 5 | -21 ± 11 | 2 ± 4 | -28 ± 18 |
| **210-215** | -1 ± 1 | 4 ± 14 | 3 ± 2 | -17 ± 7 | 1 ± 1 | -43 ± 12 | 2 ± 3 | -2 ± 6 | 3 ± 5 | -23 ± 11 | 2 ± 3 | -30 ± 17 |
| **215-220** | -1 ± 1 | 9 ± 17 | 2 ± 2 | -17 ± 7 | 1 ± 1 | -43 ± 12 | 2 ± 3 | -3 ± 6 | 2 ± 5 | -23 ± 11 | 2 ± 3 | -29 ± 17 |
| **220-225** | -2 ± 1 | -8 ± 7 | 1 ± 2 | -16 ± 6 | -1 ± 1 | -42 ± 13 | -1 ± 3 | -2 ± 8 | 1 ± 5 | -25 ± 12 | 3 ± 3 | -31 ± 17 |
| **225-230** | -1 ± 1 | -11 ± 7 | 2 ± 2 | -17 ± 6 | 1 ± 1 | -39 ± 13 | -1 ± 3 | -2 ± 3 | 1 ± 5 | -23 ± 12 | 2 ± 3 | -33 ± 16 |
| **230-235** | -1 ± 2 | -12 ± 6 | 2 ± 2 | -15 ± 5 | 2 ± 1 | -38 ± 12 | 0 ± 3 | -1 ± 10 | -1 ± 4 | -22 ± 12 | 1 ± 3 | -32 ± 17 |
| **235-240** | -4 ± 1 | -16 ± 4 | 0 ± 1 | -14 ± 8 | 1 ± 1 | -34 ± 11 | 0 ± 3 | 4 ± 16 | -1 ± 4 | -22 ± 12 | 1 ± 3 | -32 ± 17 |
| **240-245** | -4 ± 1 | -14 ± 5 | 1 ± 1 | -16 ± 7 | 0 ± 1 | -38 ± 11 | 0 ± 3 | 3 ± 12 | -1 ± 5 | -24 ± 12 | 3 ± 3 | -29 ± 19 |
| **245-250** | -2 ± 1 | -13 ± 5 | 1 ± 1 | -17 ± 7 | 0 ± 1 | -34 ± 12 | 0 ± 2 | 7 ± 12 | -2 ± 5 | -22 ± 11 | 2 ± 3 | -29 ± 18 |
| **250-255** | -1 ± 1 | -14 ± 5 | 0 ± 2 | -17 ± 7 | 0 ± 1 | -34 ± 12 | 0 ± 3 | 3 ± 10 | -2 ± 4 | -22 ± 11 | 1 ± 3 | -27 ± 19 |
| **255-260** | -3 ± 1 | -16 ± 4 | 1 ± 1 | -16 ± 7 | 0 ± 1 | -35 ± 11 | 1 ± 3 | 1 ± 9 | -2 ± 4 | -23 ± 10 | 1 ± 3 | -26 ± 20 |
| **260-265** | -3 ± 1 | -13 ± 5 | 0 ± 2 | -16 ± 7 | -1 ± 1 | -33 ± 11 | 1 ± 3 | -1 ± 8 | -3 ± 4 | -21 ± 10 | 1 ± 3 | -27 ± 20 |
| **265-270** | -2 ± 1 | 7 ± 23 | 0 ± 2 | -15 ± 6 | 0 ± 1 | -34 ± 11 | 1 ± 3 | -4 ± 8 | -2 ± 4 | -23 ± 10 | 1 ± 3 | -28 ± 20 |
| **270-275** | -2 ± 1 | -11 ± 6 | 1 ± 1 | -17 ± 6 | 1 ± 1 | -34 ± 11 | -2 ± 3 | 0 ± 9 | -2 ± 4 | -23 ± 9 | 1 ± 3 | -28 ± 20 |
| **275-280** | -3 ± 1 | -13 ± 5 | 0 ± 2 | -17 ± 6 | 0 ± 1 | -30 ± 11 | 1 ± 3 | -1 ± 10 | -3 ± 5 | -20 ± 9 | 0 ± 3 | -29 ± 18 |
| **280-285** | -5 ± 5 | -15 ± 5 | -1 ± 1 | -15 ± 6 | 0 ± 1 | -31 ± 11 | 0 ± 3 | -4 ± 8 | -4 ± 5 | -20 ± 9 | 1 ± 3 | -30 ± 18 |
| **285-290** | -3 ± 0 | -12 ± 6 | 0 ± 2 | -16 ± 6 | 0 ± 1 | -31 ± 11 | -1 ± 3 | -2 ± 1 | -3 ± 5 | -21 ± 9 | 1 ± 3 | -31 ± 17 |
| **290-295** | -5 ± 2 | -7 ± 4 | 0 ± 1 | -15 ± 5 | 0 ± 1 | -32 ± 11 | -2 ± 3 | -6 ± 6 | -4 ± 5 | -21 ± 10 | 1 ± 4 | -31 ± 17 |
| **295-300** | -4 ± 1 | -8 ± 4 | 0 ± 1 | -18 ± 7 | 0 ± 1 | -30 ± 12 | -3 ± 3 | -3 ± 4 | -4 ± 5 | -20 ± 10 | 2 ± 4 | -31 ± 17 |
|  |  |  |  |  |  |  |  |  |  |  |  |  |
