# Supplementary material for: Role of autonomic receptors in ethyl ferulate-induced cardiovascular effects in normotensive and hypertensive female rats
Source: Pflugers Arch. 2026 Apr 25;478(5):44. doi: 10.1007/s00424-026-03170-3 (PMC13110241; doi:10.1007/s00424-026-03170-3)
Supplement: Supplementary file 13 — Supplementary Material 8 Changes in MAP and HR induced by EF in Wistar and SHR females one minute after pre-treatment with or without atenolol. Data are expressed as mean ± SEM.(DOCX 1.97 MB) [file 424_2026_3170_MOESM8_ESM.docx]

Supplementary Table 3

|  | **Atenolol**  **EF 7.5 mg/kg Wistar** | | **Atenolol**  **EF 15 mg/kg Wistar** | | **Atenolol**  **EF 30 mg/kg Wistar** | | **Atenolol**  **EF 7.5 mg/kg SHR** | | **Atenolol**  **EF 15 mg/kg**  **SHR** | | **Atenolol**  **EF 30 mg/kg**  **SHR** | |
| --- | --- | --- | --- | --- | --- | --- | --- | --- | --- | --- | --- | --- |
| **Time (s)** | **MAP**  **(mmHg)** | **HR**  **(bpm)** | **MAP**  **(mmHg)** | **HR**  **(bpm)** | **MAP**  **(mmHg)** | **HR**  **(bpm)** | **MAP**  **(mmHg)** | **HR**  **(bpm)** | **MAP**  **(mmHg)** | **HR**  **(bpm)** | **MAP**  **(mmHg)** | **HR**  **(bpm)** |
| **60-65** | 5 ± 2 | 5 ± 7 | 1 ± 1 | -20 ± 9 | 0 ± 1 | -48 ± 15 | 15 ± 3 | -11 ± 2 | 19 ± 4 | -3 ± 7 | 4 ± 7 | -3 ± 21 |
| **65-70** | 5 ± 2 | 3 ± 8 | 1 ± 1 | -22 ± 11 | 0 ± 1 | -50 ± 17 | 14 ± 2 | -7 ± 2 | 22 ± 5 | -3 ± 5 | 5 ± 7 | -3 ± 21 |
| **70-75** | 4 ± 3 | 2 ± 7 | 1 ± 1 | -23 ± 12 | -1 ± 1 | -52 ± 18 | 14 ± 3 | -9 ± 4 | 21 ± 5 | -3 ± 6 | 6 ± 7 | 5 ± 19 |
| **75-80** | 5 ± 2 | 0 ± 8 | 1 ± 1 | -22 ± 9 | -1 ± 1 | -54 ± 19 | 14 ± 3 | -11 ± 5 | 18 ± 5 | -4 ± 6 | 7 ± 6 | -9 ± 19 |
| **80-85** | 4 ± 2 | -2 ± 5 | 0 ± 1 | -22 ± 9 | -2 ± 1 | -52 ± 19 | 14 ± 2 | -10 ± 5 | 18 ± 5 | -3 ± 6 | 9 ± 6 | -12 ± 17 |
| **85-90** | 4 ± 3 | -5 ± 3 | 0 ± 1 | -25 ± 11 | -1 ± 1 | -52 ± 19 | 15 ± 2 | -13 ± 3 | 17 ± 5 | -4 ± 5 | 9 ± 6 | -17 ± 15 |
| **90-95** | 4 ± 3 | -7 ± 3 | 0 ± 1 | -27 ± 12 | -2 ± 1 | -49 ± 20 | 14 ± 2 | -12 ± 3 | 17 ± 5 | -4 ± 5 | 10 ± 5 | -23 ± 17 |
| **95-100** | 4 ± 3 | -5 ± 4 | 0 ± 1 | -24 ± 9 | -2 ± 1 | -48 ± 20 | 12 ± 3 | -10 ± 4 | 17 ± 5 | -5 ± 4 | 11 ± 6 | -25 ± 17 |
| **100-105** | 4 ± 3 | -4 ± 4 | 0 ± 1 | -28 ± 11 | -2 ± 1 | -49 ± 21 | 13 ± 2 | -10 ± 4 | 16 ± 5 | -4 ± 5 | 12 ± 6 | -27 ± 16 |
| **105-110** | 4 ± 3 | -3 ± 4 | 0 ± 1 | -26 ± 11 | -2 ± 1 | -50 ± 21 | 13 ± 2 | -10 ± 4 | 16 ± 5 | -1 ± 5 | 12 ± 6 | -31 ± 15 |
| **110-115** | 3 ± 3 | -5 ± 3 | 0 ± 1 | -24 ± 10 | -2 ± -2 | -50 ± 21 | 7 ± 5 | -14 ± 7 | 17 ± 5 | 3 ± 5 | 14 ± 6 | -33 ± 13 |
| **115-120** | 3 ± 3 | -3 ± 5 | 0 ± 1 | -25 ± 11 | -2 ± 1 | -50 ± 21 | 8 ± 2 | -10 ± 5 | 14 ± 4 | -3 ± 4 | 14 ± 7 | -35 ± 11 |
| **120-125** | 4 ± 2 | 1 ± 5 | 0 ± 1 | -25 ± 11 | -2 ± 1 | -49 ± 20 | 10 ± 2 | -7 ± 7 | 13 ± 5 | -2 ± 5 | 15 ± 7 | -37 ± 9 |
| **125-130** | 4 ± 3 | 0 ± 6 | 0 ± 1 | -25 ± 11 | -2 ± 1 | -50 ± 21 | 13 ± 2 | -9 ± 8 | 12 ± 5 | -2 ± 6 | 13 ± 8 | -45 ± 6 |
| **130-135** | 3 ± 3 | -3 ± 6 | -1 ± 0 | -23 ± 8 | -2 ± 1 | -49 ± 21 | 11 ± 1 | -6 ± 7 | 13 ± 5 | -6 ± 7 | 15 ± 8 | -37 ± 12 |
| **135-140** | 2 ± 3 | -4 ± 6 | -1 ± 1 | -24 ± 9 | -2 ± 1 | -49 ± 21 | 10 ± 2 | -3 ± 10 | 14 ± 6 | -10 ± 9 | 14 ± 8 | -46 ± 5 |
| **140-145** | 3 ± 3 | -1 ± 6 | -1 ± 1 | -26 ± 12 | -2 ± 1 | -50 ± 21 | 9 ± 2 | -5 ± 9 | 12 ± 6 | -5 ± 7 | 15 ± 7 | -49 ± 6 |
| **145-150** | 3 ± 3 | -1 ± 7 | 0 ± 1 | -22 ± 9 | -1 ± 1 | -49 ± 21 | 10 ± 2 | -6 ± 8 | 9 ± 6 | -2 ± 6 | 15 ± 8 | -48 ± 5 |
| **150-155** | 2 ± 3 | -2 ± 6 | -1 ± 1 | -21 ± 8 | -1 ± 1 | -49 ± 21 | 10 ± 2 | -8 ± 9 | 9 ± 6 | -2 ± 5 | 14 ± 7 | -48 ± 6 |
| **155-160** | 3 ± 3 | -1 ± 6 | -1 ± 1 | -24 ± 10 | -1 ± 1 | -49 ± 21 | 10 ± 2 | -8 ± 9 | 10 ± 6 | -6 ± 6 | 14 ± 7 | -46 ± 6 |
| **160-165** | 2 ± 3 | -2 ± 5 | -1 ± 1 | -21 ± 9 | -1 ± 1 | -47 ± 21 | 6 ± 2 | -5 ± 7 | 9 ± 5 | -5 ± 6 | 13 ± 7 | -45 ± 5 |
| **165-170** | 2 ± 3 | -4 ± 5 | -1 ± 1 | -22 ± 10 | 0 ± 1 | -47 ± 21 | 7 ± 3 | -2 ± 7 | 9 ± 5 | 0 ± 5 | 11 ± 7 | -47 ± 5 |
| **170-175** | 2 ± 3 | -5 ± 4 | -1 ± 1 | -22 ± 11 | 0 ± 0 | -47 ± 21 | 4 ± 3 | -8 ± 9 | 10 ± 6 | -3 ± 5 | 11 ± 7 | -43 ± 3 |
| **175-180** | 2 ± 3 | -4 ± 5 | -1 ± 1 | -23 ± 11 | -1 ± 1 | -48 ± 20 | 7 ± 3 | -4 ± 8 | 7 ± 6 | 0 ± 5 | 7 ± 7 | -44 ± 5 |
| **180-185** | 2 ± 3 | -5 ± 5 | -1 ± 1 | -23 ± 10 | 0 ± 1 | -49 ± 21 | 7 ± 2 | -4 ± 9 | 7 ± 6 | -1 ± 6 | 7 ± 7 | -40 ± 3 |
| **185-190** | 2 ± 3 | -2 ± 5 | 0 ± 1 | -17 ± 7 | -2 ± 1 | -48 ± 20 | 7 ± 3 | -2 ± 9 | 8 ± 5 | 0 ± 7 | 7 ± 7 | -36 ± 2 |
| **190-195** | 2 ± 3 | -1 ± 5 | 0 ± 1 | -18 ± 7 | -1 ± 1 | -48 ± 21 | 6 ± 3 | -2 ± 8 | 6 ± 5 | 7 ± 6 | 7 ± 6 | -27 ± 8 |
| **195-200** | 2 ± 3 | -2 ± 5 | -1 ± 1 | -19 ± 7 | -2 ± 1 | -48 ± 20 | 8 ± 3 | -3 ± 9 | 5 ± 5 | 4 ± 7 | 5 ± 7 | -35 ± 3 |
| **200-205** | 2 ± 3 | 0 ± 5 | -1 ± 1 | -18 ± 7 | -1 ± 1 | -47 ± 20 | 6 ± 4 | -1 ± 9 | 4 ± 5 | 0 ± 9 | 5 ± 7 | -37 ± 4 |
| **205-210** | 2 ± 3 | -2 ± 5 | -1 ± 1 | -16 ± 6 | -2 ± 1 | -49 ± 20 | 3 ± 3 | -10 ± 7 | 4 ± 5 | 2 ± 9 | 5 ± 7 | -36 ± 6 |
| **210-215** | 2 ± 3 | -3 ± 7 | -2 ± 1 | -15 ± 7 | -2 ± 1 | -48 ± 20 | 3 ± 3 | -5 ± 4 | 3 ± 5 | 6 ± 10 | 5 ± 7 | -36 ± 6 |
| **215-220** | 2 ± 3 | -3 ± 7 | -1 ± 1 | -13 ± 6 | -2 ± 1 | -48 ± 20 | 4 ± 3 | -5 ± 3 | 3 ± 6 | 10 ± 8 | 5 ± 7 | -34 ± 4 |
| **220-225** | 2 ± 3 | -1 ± 6 | -1 ± 1 | -14 ± 8 | -2 ± 1 | -49 ± 20 | 5 ± 4 | -9 ± 4 | 3 ± 6 | 9 ± 6 | 4 ± 6 | -32 ± 5 |
| **225-230** | 1 ± 3 | 0 ± 7 | -1 ± 1 | -15 ± 8 | -2 ± 1 | -48 ± 20 | 4 ± 3 | -5 ± 2 | 5 ± 5 | 11 ± 8 | 3 ± 7 | -34 ± 7 |
| **230-235** | 2 ± 3 | 0 ± 6 | -2 ± 1 | -15 ± 9 | -2 ± -2 | -46 ± 20 | 5 ± 3 | -8 ± 3 | 4 ± 6 | 3 ± 10 | 3 ± 7 | -35 ± 5 |
| **235-240** | 2 ± 4 | -1 ± 6 | -2 ± 1 | -15 ± 8 | -3 ± 1 | -46 ± 20 | 4 ± 3 | -9 ± 4 | 4 ± 5 | 3 ± 11 | 3 ± 6 | -40 ± 5 |
| **240-245** | 2 ± 3 | 1 ± 6 | -2 ± 1 | -15 ± 10 | -3 ± 1 | -47 ± 20 | 3 ± 3 | -8 ± 4 | 4 ± 5 | 2 ± 10 | 4 ± 6 | -37 ± 5 |
| **245-250** | 2 ± 3 | 2 ± 5 | -3 ± 1 | -13 ± 11 | -3 ± 1 | -45 ± 20 | 4 ± 4 | -7 ± 3 | 8 ± 4 | 0 ± 9 | 6 ± 5 | -38 ± 7 |
| **250-255** | 1 ± 3 | 1 ± 5 | -2 ± 1 | -10 ± 11 | -3 ± 1 | -45 ± 20 | 1 ± 4 | -6 ± 4 | 8 ± 4 | 0 ± 10 | 6 ± 6 | -27 ± 10 |
| **255-260** | 2 ± 3 | 1 ± 7 | -3 ± 1 | -9 ± 11 | -2 ± 1 | -44 ± 19 | 0 ± 5 | -4 ± 4 | 7 ± 4 | 5 ± 8 | 7 ± 6 | -31 ± 7 |
| **260-265** | 3 ± 4 | 2 ± 8 | -2 ± 1 | -8 ± 11 | -3 ± 1 | -43 ± 19 | 2 ± 2 | -10 ± 6 | 7 ± 4 | 3 ± 9 | 6 ± 5 | -28 ± 7 |
| **265-270** | 2 ± 3 | 2 ± 7 | -3 ± 1 | -8 ± 11 | -2 ± 1 | -35 ± 13 | 2 ± 3 | -4 ± 2 | 8 ± 4 | 3 ± 10 | 4 ± 5 | -24 ± 7 |
| **270-275** | 2 ± 3 | 2 ± 6 | -3 ± 2 | -8 ± 10 | -4 ± 1 | -40 ± 17 | 2 ± 3 | -5 ± 2 | 6 ± 2 | 6 ± 10 | 5 ± 5 | -24 ± 8 |
| **275-280** | 1 ± 3 | 3 ± 6 | -3 ± 2 | -5 ± 9 | -4 ± 1 | -40 ± 17 | 2 ± 4 | -5 ± 1 | 7 ± 3 | 6 ± 10 | 4 ± 5 | -22 ± 8 |
| **280-285** | 2 ± 4 | 4 ± 7 | -4 ± 2 | -5 ± 10 | -4 ± 2 | -34 ± 13 | 3 ± 3 | -10 ± 6 | 7 ± 3 | 6 ± 10 | 4 ± 6 | -23 ± 8 |
| **285-290** | 0 ± 3 | 4 ± 6 | -4 ± 2 | -6 ± 10 | -5 ± 2 | -37 ± 15 | 1 ± 2 | -6 ± 5 | 6 ± 4 | 6 ± 9 | 4 ± 6 | -24 ± 8 |
| **290-295** | 0 ± 3 | 4 ± 6 | -4 ± 3 | -8 ± 10 | -6 ± -6 | -40 ± 16 | 0 ± 3 | -6 ± 5 | 5 ± 4 | 7 ± 9 | 4 ± 5 | -26 ± 6 |
| **295-300** | 2 ± 4 | 2 ± 5 | -5 ± 3 | -7 ± 10 | -6 ± 3 | -41 ± 17 | -2 ± 5 | -6 ± 6 | 3 ± 3 | 9 ± 10 | 2 ± 5 | -30 ± 3 |
|  |  |  |  |  |  |  |  |  |  |  |  |  |
