# Supplementary material for: Role of autonomic receptors in ethyl ferulate-induced cardiovascular effects in normotensive and hypertensive female rats
Source: Pflugers Arch. 2026 Apr 25;478(5):44. doi: 10.1007/s00424-026-03170-3 (PMC13110241; doi:10.1007/s00424-026-03170-3)
Supplement: Supplementary file 14 — Supplementary Material 9 Changes in MAP and HR induced by EF in Wistar and SHR females one minute after pre-treatment with or without hexamethonium. Data are expressed as mean ± SEM.(DOCX 1.97 MB) [file 424_2026_3170_MOESM9_ESM.docx]

Supplementary Table 4

|  | **Hexamethonium**  **EF 7.5 mg/kg Wistar** | | **Hexamethonium**  **EF 15 mg/kg Wistar** | | **Hexamethonium**  **EF 30 mg/kg Wistar** | | **Hexamethonium**  **EF 7.5 mg/kg SHR** | | **Hexamethonium**  **EF 15 mg/kg**  **SHR** | | **Hexamethonium**  **EF 30 mg/kg**  **SHR** | |
| --- | --- | --- | --- | --- | --- | --- | --- | --- | --- | --- | --- | --- |
| **Time (s)** | **MAP**  **(mmHg)** | **HR**  **(bpm)** | **MAP**  **(mmHg)** | **HR**  **(bpm)** | **MAP**  **(mmHg)** | **HR**  **(bpm)** | **MAP**  **(mmHg)** | **HR**  **(bpm)** | **MAP**  **(mmHg)** | **HR**  **(bpm)** | **MAP**  **(mmHg)** | **HR**  **(bpm)** |
| **60-65** | 12 ± 5 | -6 ± 11 | 4 ± 3 | -49 ± 12 | 11 ±5 | -67 ±17 | 15 ± 3 | -6 ± 3 | 18 ± 4 | -23 ± 11 | 18 ± 4 | -37 ± 16 |
| **65-70** | 10 ± 5 | -4 ± 11 | 4 ± 3 | -49 ± 12 | 13 ± 5 | -75 ±15 | 15 ± 3 | -6 ± 2 | 18 ± 4 | -21 ± 9 | 18 ± 4 | -40 ± 17 |
| **70-75** | 10 ± 5 | -3 ± 11 | 5 ± 3 | -51 ± 12 | 13 ± 5 | -76 ± 14 | 15 ± 3 | -6 ± 3 | 19 ± 4 | -19 ± 8 | 19 ± 4 | -40 ± 17 |
| **75-80** | 11 ± 5 | -4 ± 11 | 5 ± 3 | -53 ± 13 | 12 ± 5 | -75 ± 14 | 16 ± 3 | -5 ± 3 | 20 ± 4 | -17 ± 8 | 18 ± 4 | -41 ± 17 |
| **80-85** | 12 ± 5 | -7 ± 10 | 6 ± 3 | -51 ± 13 | 13 ± 5 | -75 ± 13 | 17 ± 3 | -5 ± 3 | 21 ± 4 | -18 ± 9 | 18 ± 4 | -39 ± 17 |
| **85-90** | 12 ± 4 | -7 ± 11 | 4 ± 3 | -48 ± 12 | 11 ± 5 | -73 ± 13 | 17 ± 3 | -6 ± 3 | 21 ± 4 | -17 ± 10 | 17 ± 4 | -39 ± 18 |
| **90-95** | 12 ± 5 | -8 ± 10 | 5 ± 3 | -50 ± 13 | 10 ± 4 | -72 ± 13 | 16 ± 3 | -7 ± 3 | 22 ± 4 | -24 ± 9 | 17 ± 4 | -38 ± 18 |
| **95-100** | 11 ± 5 | -5 ± 11 | 5 ± 3 | -47 ± 12 | 10 ± 4 | -70 ± 14 | 17 ± 3 | -8 ± 3 | 21 ± 4 | -29 ± 10 | 16 ± 5 | -41 ± 18 |
| **100-105** | 10 ± 4 | -3 ± 11 | 5 ± 3 | -48 ± 12 | 10 ± 5 | -72 ± 12 | 17 ± 4 | -8 ± 3 | 21 ± 4 | -25 ± 10 | 18 ± 5 | -44 ± 17 |
| **105-110** | 10 ± 4 | -2 ± 10 | 5 ± 3 | -49 ± 12 | 10 ± 4 | -71 ± 12 | 16 ± 4 | -9 ± 3 | 22 ± 4 | -22 ± 11 | 19 ± 5 | -43 ± 16 |
| **110-115** | 8 ± 3 | 1 ± 10 | 4 ± 2 | -48 ± 12 | 9 ± 4 | -66 ± 14 | 16 ± 4 | -8 ± 3 | 23 ± 4 | -23 ± 10 | 15 ± 5 | -43 ± 16 |
| **115-120** | 10 ± 3 | 3 ± 10 | 4 ± 2 | -48 ± 12 | 8 ± 4 | -65 ± 14 | 16 ± 3 | -8 ± 4 | 23 ± 4 | -23 ± 10 | 17 ± 5 | -45 ± 18 |
| **120-125** | 10 ± 3 | 0 ± 10 | 3 ± 2 | -48 ± 11 | 9 ± 4 | -63 ± 15 | 15 ± 3 | -9 ± 3 | 23 ± 4 | -23 ± 10 | 16 ± 6 | -46 ± 16 |
| **125-130** | 10 ± 3 | 0 ± 10 | 3 ± 2 | -51 ± 12 | 10 ± 4 | -63 ± 15 | 15 ± 4 | -9 ± 3 | 22 ±45 | -22 ± 9 | 17 ± 6 | -41 ± 14 |
| **130-135** | 12 ± 2 | -1 ± 10 | 3 ± 2 | -51 ± 12 | 9 ± 4 | -62 ± 16 | 16 ± 3 | -9 ± 3 | 19 ± 5 | -15 ± 6 | 18 ± 5 | -40 ± 14 |
| **135-140** | 11 ± 5 | -5 ± 11 | 4 ± 2 | -51 ± 12 | 9 ± 4 | -62 ± 16 | 16 ± 3 | -10 ± 3 | 18 ± 5 | -13 ± 6 | 19 ± 6 | -42 ± 14 |
| **140-145** | 11 ± 2 | -1 ± 10 | 3 ± 2 | -51 ± 12 | 9 ± 4 | -63 ± 15 | 16 ± 3 | -10 ± 3 | 18 ± 5 | -13 ± 7 | 19 ± 6 | -39 ± 15 |
| **145-150** | 11 ± 2 | 0 ± 10 | 4 ± 2 | -52 ± 12 | 8 ± 4 | -64 ± 15 | 14 ± 3 | -9 ± 4 | 18 ± 5 | -13 ± 7 | 20 ± 6 | -41 ± 15 |
| **150-155** | 11 ± 3 | 0 ± 10 | 4 ± 2 | -52 ± 12 | 8 ± 3 | -63 ± 15 | 13 ± 4 | -9 ± 4 | 19 ± 4 | -16 ± 7 | 20 ± 6 | -39 ± 16 |
| **155-160** | 12 ± 2 | 0 ± 10 | 5 ± 2 | -51 ± 12 | 7 ± 3 | -62 ± 15 | 14 ± 3 | -10 ± 3 | 19 ± 4 | -16 ± 7 | 19 ± 6 | -42 ± 15 |
| **160-165** | 11 ± 3 | 0 ± 10 | 4 ± 1 | -50 ± 12 | 7 ± 3 | -61 ± 15 | 15 ± 3 | -10 ± 3 | 17 ± 5 | -17 ± 8 | 18 ± 6 | -41 ± 16 |
| **165-170** | 11 ± 3 | 0 ± 10 | 4 ± 1 | -50 ± 12 | 8 ± 3 | -61 ± 15 | 15 ± 3 | -8 ± 4 | 17 ± 5 | -15 ± 8 | 18 ± 6 | -41 ± 15 |
| **170-175** | 11 ± 3 | 1 ± 10 | 4 ± 1 | -48 ± 12 | 7 ± 3 | -60 ± 15 | 14 ± 4 | -8 ± 3 | 17 ± 4 | -13 ± 7 | 18 ± 6 | -39 ± 16 |
| **175-180** | 11 ± 2 | 2 ± 11 | 5 ± 1 | -48 ± 12 | 7 ± 3 | -58 ± 16 | 14 ± 3 | -10 ± 3 | 18 ± 4 | -14 ± 7 | 17 ± 6 | -38 ± 15 |
| **180-185** | 12 ± 3 | 1 ± 11 | 4 ± 1 | -49 ± 12 | 8 ± 3 | -58 ± 16 | 11 ± 4 | -10 ± 4 | 19 ± 4 | -17 ± 8 | 16 ± 7 | -34 ± 16 |
| **185-190** | 13 ± 3 | 0 ± 11 | 5 ± 2 | -49 ± 12 | 8 ± 3 | -60 ± 15 | 13 ± 3 | -10 ± 4 | 18 ± 4 | -18 ± 7 | 16 ± 7 | -39 ± 14 |
| **190-195** | 12 ± 3 | 1 ± 12 | 5 ± 2 | -52 ± 11 | 8 ± 3 | -58 ± 15 | 13 ± 3 | -10 ± 4 | 16 ± 4 | -14 ± 5 | 16 ± 7 | -38 ± 15 |
| **195-200** | 11 ± 2 | 1 ± 11 | 5 ± 1 | -49 ± 12 | 8 ± 3 | -63 ± 15 | 13 ± 3 | -10 ± 4 | 16 ± 4 | -16 ± 6 | 17 ± 6 | -41 ± 16 |
| **200-205** | 10 ± 2 | 7 ± 10 | 5 ± 1 | -50 ± 12 | 8 ± 3 | -62 ± 16 | 13 ± 3 | -11 ± 3 | 16 ± 5 | -16 ± 6 | 17 ± 7 | -41 ± 17 |
| **205-210** | 10 ± 2 | 7 ± 11 | 4 ± 1 | -49 ± 12 | 8 ± 3 | -58 ± 16 | 12 ± 3 | -9 ± 3 | 15 ± 4 | -15 ± 8 | 18 ± 6 | -42 ± 17 |
| **210-215** | 9 ± 2 | 6 ± 11 | 4 ± 1 | -48 ± 12 | 8 ± 3 | -57 ± 16 | 11 ± 4 | -9 ± 3 | 15 ± 4 | -15 ± 8 | 17 ± 6 | -39 ± 17 |
| **215-220** | 10 ± 3 | 4 ± 11 | 4 ± 1 | -49 ± 12 | 8 ± 3 | -56 ± 15 | 12 ± 3 | -9 ± 3 | 16 ± 4 | -15 ± 8 | 17 ± 6 | -41 ± 17 |
| **220-225** | 10 ± 2 | 5 ± 11 | 4 ± 1 | -48 ± 12 | 6 ± 3 | -51 ± 16 | 11 ± 3 | -9 ± 3 | 17 ± 4 | -18 ± 7 | 17 ± 6 | -40 ± 16 |
| **225-230** | 11 ± 3 | 4 ± 11 | 4 ± 1 | -44 ± 11 | 6 ± 3 | -53 ± 16 | 11 ± 4 | -9 ± 3 | 15 ± 4 | -14 ± 7 | 18 ± 6 | -43 ± 16 |
| **230-235** | 12 ± 3 | 4 ± 12 | 4 ± 1 | -45 ± 11 | 6 ± 3 | -52 ± 15 | 10 ± 3 | -9 ± 3 | 13 ± 3 | -11 ± 6 | 17 ± 6 | -40 ± 15 |
| **235-240** | 11 ± 2 | 4 ± 12 | 3 ± 1 | -42 ± 12 | 6 ± 3 | -52 ± 15 | 11 ± 3 | -9 ± 3 | 14 ± 4 | -11 ± 5 | 17 ± 6 | -41 ± 14 |
| **240-245** | 10 ± 2 | 6 ± 11 | 3 ± 1 | -45 ± 11 | 4 ± 3 | -49 ± 14 | 11 ± 3 | -10 ± 3 | 14 ± 4 | -12 ± 6 | 17 ± 6 | -40 ± 14 |
| **245-250** | 10 ± 3 | 10 ± 12 | 4 ± 1 | -42 ± 11 | 5 ± 3 | -48 ± 14 | 10 ± 3 | -10 ± 3 | 15 ± 4 | -13 ± 6 | 17 ± 6 | -42 ± 15 |
| **250-255** | 10 ± 2 | 10 ± 11 | 2 ± 1 | -40 ± 11 | 5 ± 3 | -51 ± 15 | 11 ± 3 | -10 ± 3 | 14 ± 4 | -10 ± 5 | 16 ± 7 | -38 ± 15 |
| **255-260** | 9 ± 3 | 8 ± 11 | 3 ± 1 | -39 ± 11 | 4 ± 3 | -52 ± 15 | 8 ± 4 | -8 ± 3 | 15 ± 4 | -13 ± 6 | 17 ± 7 | -38 ± 15 |
| **260-265** | 9 ± 3 | 10 ± 11 | 3 ± 1 | -40 ± 11 | 5 ± 3 | -52 ± 15 | 9 ± 4 | -9 ± 3 | 14 ± 4 | -13 ± 6 | 16 ± 7 | -35 ± 16 |
| **265-270** | 9 ± 3 | 10 ± 11 | 2 ± 1 | -40 ± 11 | 4 ± 3 | -50 ± 15 | 9 ± 3 | -9 ± 3 | 13 ± 4 | -12 ± 5 | 15 ± 7 | -38 ± 15 |
| **270-275** | 9 ± 3 | 10 ± 10 | 2 ± 1 | -37 ± 11 | 5 ± 3 | -49 ± 15 | 9 ± 3 | -8 ± 3 | 12 ± 4 | -13 ± 5 | 15 ± 7 | -34 ± 15 |
| **275-280** | 9 ± 4 | 11 ± 10 | 3 ± 1 | -36 ± 10 | 5 ± 3 | -51 ± 14 | 9 ± 3 | -8 ± 3 | 12 ± 4 | -14 ± 6 | 21 ± 9 | -40 ± 14 |
| **280-285** | 9 ± 3 | 11 ± 10 | 2 ± 1 | -34 ± 11 | 5 ± 3 | -50 ± 14 | 8 ± 4 | -5 ± 4 | 12 ± 5 | -15 ± 7 | 18 ± 9 | -38 ± 14 |
| **285-290** | 10 ± 3 | 11 ± 10 | 3 ± 1 | -34 ± 11 | 5 ± 3 | -49 ± 13 | 7 ± 4 | -6 ± 3 | 12 ± 5 | -13 ± 7 | 19 ± 9 | -37 ± 13 |
| **290-295** | 10 ± 4 | 7 ± 11 | 2 ± 1 | -35 ± 10 | 4 ± 2 | -48 ± 13 | 7 ± 4 | -6 ± 3 | 13 ± 5 | -14 ± 7 | 17 ± 9 | -34 ± 13 |
| **295-300** | 10 ± 4 | 4 ± 11 | 2 ± 1 | -36 ± 10 | 4 ± 2 | -47 ± 13 | 7 ± 4 | -6 ± 3 | 13 ± 5 | -12 ± 5 | 18 ± 9 | -36 ± 12 |
|  |  |  |  |  |  |  |  |  |  |  |  |  |
