# Supplementary material for: Role of autonomic receptors in ethyl ferulate-induced cardiovascular effects in normotensive and hypertensive female rats
Source: Pflugers Arch. 2026 Apr 25;478(5):44. doi: 10.1007/s00424-026-03170-3 (PMC13110241; doi:10.1007/s00424-026-03170-3)
Supplement: Supplementary file 15 — Supplementary Material 10 Changes in MAP and HR induced by EF in SHR females one minute after pre-treatment with or without L-NAME. Data are expressed as mean ± SEM.(DOCX 1.96 MB) [file 424_2026_3170_MOESM10_ESM.docx]

Supplementary Table 5

|  | **L-NAME**  **EF 7.5 mg/kg SHR** | | **L-NAME**  **EF 15 mg/kg**  **SHR** | | **L-NAME**  **EF 30 mg/kg**  **SHR** | |
| --- | --- | --- | --- | --- | --- | --- |
| **Time (s)** | **MAP**  **(mmHg)** | **HR**  **(bpm)** | **MAP**  **(mmHg)** | **HR**  **(bpm)** | **MAP**  **(mmHg)** | **HR**  **(bpm)** |
| **60-65** | 4 ± 2 | 9 ± 6 | 9 ± 5 | -28 ± 23 | 19 ± 8 | 14 ± 26 |
| **65-70** | -5 ± 3 | -17 ± 4 | 9 ± 5 | -29 ± 19 | 18 ± 8 | 14 ± 27 |
| **70-75** | -5 ± 3 | -18 ± 3 | 8 ± 4 | -29 ± 18 | 18 ± 8 | 15 ± 27 |
| **75-80** | -4 ± 2 | -23 ± 4 | 9 ± 4 | -27 ± 14 | 19 ± 8 | 27 ± 27 |
| **80-85** | -5 ± 2 | -29 ± 3 | 10 ± 4 | -26 ± 13 | 17 ± 7 | 30 ± 29 |
| **85-90** | -5 ± 2 | -24 ± 5 | 9 ± 4 | -41 ± 16 | 16 ± 6 | 13 ± 22 |
| **90-95** | -4 ± 2 | -24 ± 6 | 10 ± 4 | -43 ± 15 | 16 ± 6 | -3 ± 26 |
| **95-100** | -4 ± 2 | -22 ± 6 | 11 ± 4 | -39 ± 11 | 15 ± 6 | -10 ± 20 |
| **100-105** | -6 ± 2 | -20 ± 6 | 10 ± 4 | -37 ± 11 | 16 ± 5 | -13 ± 22 |
| **105-110** | -5 ± 2 | -21 ± 7 | 10 ± 4 | -43 ± 13 | 15 ± 5 | -12 ± 23 |
| **110-115** | -5 ± 2 | -22 ± 8 | 10 ± 4 | -34 ± 11 | 14 ± 4 | -13 ± 21 |
| **115-120** | -5 ± 2 | -23 ± 8 | 9 ± 4 | -33 ± 11 | 13 ± 3 | -16 ± 17 |
| **120-125** | 4 ± 1 | 25 ± 7 | 9 ± 4 | -37 ± 11 | 13 ± 3 | -26 ± 15 |
| **125-130** | -4 ± 1 | -28 ± 4 | 7 ± 4 | -18 ± 24 | 13 ± 3 | -36 ± 15 |
| **130-135** | -5 ± 2 | -33 ± 7 | 8 ± 4 | -41 ± 11 | 14 ± 3 | -37 ± 15 |
| **135-140** | -4 ± 1 | -28 ± 4 | 7 ± 4 | -58 ± 22 | 13 ± 4 | -44 ± 19 |
| **140-145** | -4 ± 1 | -31 ± 4 | 8 ± 3 | -57 ± 22 | 12 ± 3 | -39 ± 17 |
| **145-150** | -6 ± 1 | -29 ± 7 | 9 ± 3 | -54 ± 20 | 11 ± 4 | -39 ± 18 |
| **150-155** | -5 ± 1 | -25 ± 6 | 8 ± 3 | -52 ± 19 | 10 ± 4 | -40 ± 15 |
| **155-160** | -7 ± 3 | -29 ± 9 | 8 ± 3 | -43 ± 13 | 10 ± 4 | -41 ± 15 |
| **160-165** | -5 ± 2 | -27 ± 11 | 6 ± 3 | -43 ± 14 | 10 ± 3 | -40 ± 16 |
| **165-170** | -5 ± 1 | -28 ± 9 | 5 ± 3 | -36 ± 12 | 9 ± 3 | -40 ± 17 |
| **170-175** | -5 ± 1 | -28 ± 8 | 5 ± 2 | -48 ± 16 | 6 ± 4 | -35 ± 17 |
| **175-180** | -5 ± 1 | -29 ± 7 | 4 ± 2 | -54 ± 20 | 5 ± 3 | -35 ± 17 |
| **180-185** | 8 ± 4 | 26 ± 7 | 4 ± 2 | -55 ± 23 | 3 ± 5 | -36 ± 16 |
| **185-190** | -5 ± 1 | -24 ± 11 | 3 ± 2 | -61 ± 22 | 2 ± 4 | -34 ± 19 |
| **190-195** | -7 ± 2 | -18 ± 17 | 5 ± 2 | -55 ± 22 | 1 ± 4 | -33 ± 17 |
| **195-200** | -6 ± 2 | -15 ± 20 | 5 ± 2 | -51 ± 19 | 1 ± 3 | -30 ± 17 |
| **200-205** | -7 ± 1 | -20 ± 13 | 4 ± 2 | -49 ± 17 | -2 ± 4 | -28 ± 16 |
| **205-210** | -8 ± 2 | -16 ± 16 | 4 ± 2 | -42 v 13 | -2 ± 4 | -35 ± 18 |
| **210-215** | -7 ± 2 | -19 ± 13 | 4 ± 2 | -37 ± 10 | -3 ± 3 | -34 ± 17 |
| **215-220** | -6 ± 2 | -18 ± 13 | 3 ±2 | -35 ± 10 | -2 ± 2 | -34 ± 16 |
| **220-225** | -6 ± 1 | -22 ± 13 | 3 ± 3 | -35 ± 12 | -2 ± 2 | -33 ± 16 |
| **225-230** | -6 ± 1 | -21 ± 11 | 1 ± 3 | -36 ± 11 | -4 ± 2 | -32 ± 18 |
| **230-235** | -6 ± 1 | -19 ± 12 | 1 ± 2 | -31 ± 11 | -5 ± 2 | -33 ± 18 |
| **235-240** | -6 ± 1 | -20 ± 10 | 2 ± 2 | -27 ± 12 | -6 ± 1 | -31 ± 17 |
| **240-245** | 6 ± 1 | 9 ± 21 | 3 ± 2 | -20 ± 18 | -6 ± 4 | -26 ± 17 |
| **245-250** | -6 ± 2 | -18 ± 14 | 3 ± 2 | -23 ± 14 | -8 ± 4 | -27 ± 17 |
| **250-255** | -4 ± 2 | -14 ± 16 | 3 ± 2 | -25 ± 15 | -8 ± 3 | -30 ± 20 |
| **255-260** | -4 ± 2 | -12 ± 16 | 2 ± 2 | -33 ± 8 | -8 ± 3 | -34 ± 24 |
| **260-265** | -7 ± 2 | -16 ± 12 | 3 ± 2 | -33 ± 7 | -8 ± 2 | -30 ± 19 |
| **265-270** | -7 ± 2 | -19 ± 10 | 3 ± 3 | -15 ± 20 | -9 ± 2 | -30 ± 20 |
| **270-275** | -7 ± 2 | -25 ± 10 | 2 ± 3 | -22 ± 20 | -9 ± 2 | -28 ± 19 |
| **275-280** | -6 ± 2 | -22 ± 9 | 1 ± 3 | -21 ± 23 | -10 ± 2 | -31 ± 21 |
| **280-285** | -6 ± 2 | -22 ± 8 | 3 ± 4 | -21 ± 20 | -9 ± 3 | -25 ± 17 |
| **285-290** | -6 ± 2 | -24 ± 5 | 3 ± 4 | -22 ± 20 | -9 ± 3 | -25 ± 19 |
| **290-295** | -6 ± 2 | -23 ± 6 | 3 ± 4 | -20 ± 200 | -9 ± 3 | -23 ± 17 |
| **295-300** | -8 ± 3 | -30 ± 7 | 3 ± 4 | -21 ± 22 | -10 ± 3 | -27 ± 20 |
|  |  |  |  |  |  |  |
